# Supplementary material for: Benchmarking commonly used software suites and analysis workflows for DIA proteomics and phosphoproteomics
Source: Nat Commun. 2023 Jan 6;14:94. doi: 10.1038/s41467-022-35740-1 (PMC9822986; doi:10.1038/s41467-022-35740-1)
Supplement: Supplementary file 1 — Supplementary Information [file 41467_2022_35740_MOESM1_ESM.pdf]

Supplementary Information

Benchmarking commonly used software suites and analysis workflows for DIA proteomics and phosphoproteomics

Table of Content

Supplementary Figure 1 Number and overlap of protein and peptide entries in different libraries 3

Supplementary Figure 2 Comparison of the number of reported proteins and re-assigned proteins with different analysis workflows 5

Supplementary Figure 3 Identification of mouse peptides and the overlap of mouse proteins with different analysis workflows 6

Supplementary Figure 4 Identification of GPCR proteins and peptides with different analysis workflows 7

Supplementary Figure 5 Percentages of mouse proteins detected in different numbers of replicates under each dilution condition by different analysis workflows 8

Supplementary Figure 6 Library expansion for FDR/FNR assessment 9

Supplementary Figure 7 Comparison of three methods for protein intensity determination by each software with specific libraries for HF data 11

Supplementary Figure 8 Comparison of three methods for protein intensity determination by each software with specific libraries for TIMS data 13

Supplementary Figure 9 Quantification performance evaluation with HF data 15

Supplementary Figure 10 Quantification performance evaluation with TIMS data 17

Supplementary Figure 11 Differentially expressed protein (DEP) detection 19

Supplementary Figure 12 Sensitivity and specificity of the DEP analysis based on receiver operating characteristic (ROC) curves 20

Supplementary Figure 13 Human synthetic phosphopeptide data analysis by DIA-NN and Spectronaut with an DDA-independent library 21

Supplementary Figure 14 Phosphorylation stoichiometry measurement using a benchmark data set. 23

Supplementary Figure 15 Total phosphosite identification and quantification in TNF-α-induced phosphoproteomic analysis 25

Supplementary Figure 16 Completeness of detected phosphosites as a function of site confidence in TNF-α-induced phosphoproteomic analysis 26

Supplementary Figure 17 Comparison of DIA-NN and Spectronaut in TNF-α-induced phosphoproteome data analysis without phosphosite intensity imputation 27

Supplementary Note 1 Spectronaut 29
1.1 HF benchmark dataset 29
1.2 TIMS benchmark dataset 30
Supplementary Note 2 DIA-NN 31
2.1 Empirical error rate and quantification performance with varied filtering criteria for reports from two benchmark datasets 31
2.2 Quantification strategy 35
2.3 Filtering criteria for synthetic phosphopeptide dataset 38
Supplementary Note 3 Skyline 39
3.1 HF benchmark data with different MS2 mass error tolerances 40
3.2 TIMS benchmark data with different MS2 mass error tolerances 42
3.3 Synthetic phosphopeptide dataset with different MS2 mass error tolerances 43
Supplementary Note 4 MaxDIA 45
4.1 Quantification performance with stepped transfer q-value 45
4.2 RT and precursor intensity correlation between MaxDIA and other tools 47
4.3 Improvement of MaxDIA in a recently released version 48
Supplementary Note 5 Statistics for differentially expressed protein detection 49
5.1 Levene’s test for homoscedasticity 49
5.2 Fold-change threshold and statistical test 49
5.3 Comparison of Student’s t-test, Welch's t-test, SAM and Limma 50

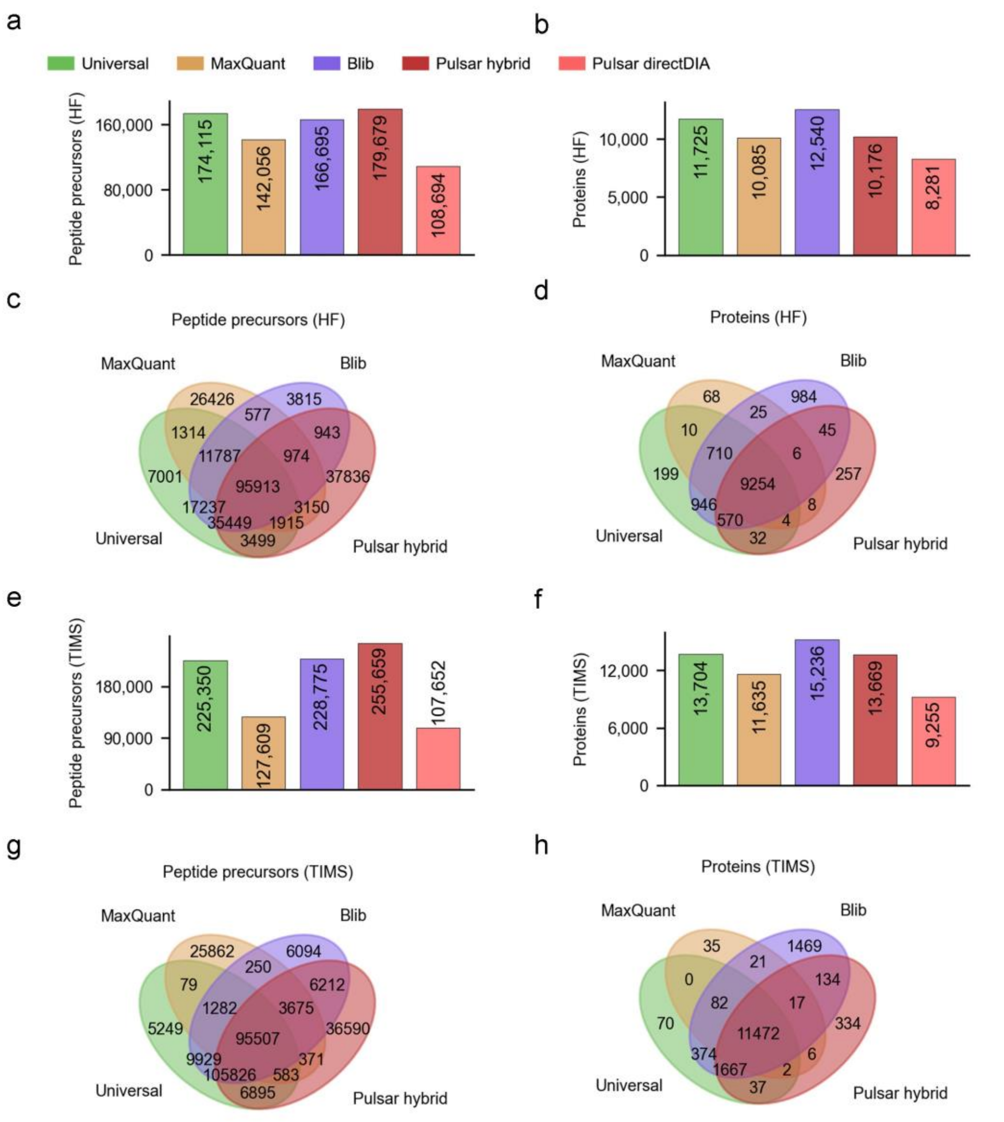

i

in silico library	Number
Total mouse and yeast proteins	23,812
Total mouse and yeast peptide sequences	1,529,467

Supplementary Figure 1 Number and overlap of protein and peptide entries in different libraries

a, b, Number of proteins **(a)** and peptide precursors **(b)** in different libraries built from HF data. **c, d,** Overlap of proteins **(c)** and peptide precursors **(d)** of the universal library and three software-specific DDA-dependent libraries built from HF data. Numbers of re-assigned proteins based on razor protein inference are used. **e, f, g, h,** Same as a, b, c, d but from TIMS data. **i,** Number of mouse and yeast protein entries in two *in silico* libraries.

Peptides are yielded according to the digestion rules of trypsin/P as enzyme, peptide length from 7 to 30, and max missed cleavage of 1.

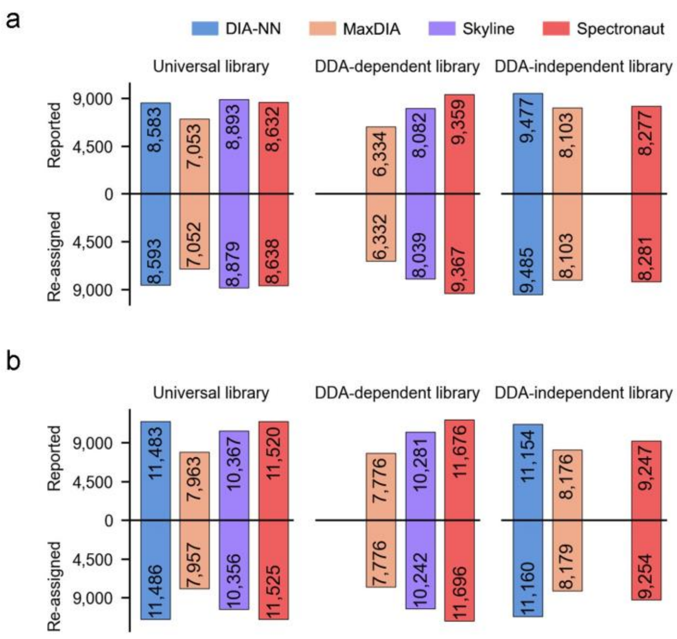

Supplementary Figure 2 Comparison of the number of reported proteins and re-assigned proteins with different analysis workflows

a, Result from HF data. **b,** Result from TIMS data. Reported proteins were those directly reported in search results, and re-assigned proteins were obtained based on razor protein inference to remove redundant identifications. All four tools were set to do protein grouping by themselves, and Skyline retained the pre-assigned proteins from the universal library. Relaxed protein inference was enabled in DIA-NN.

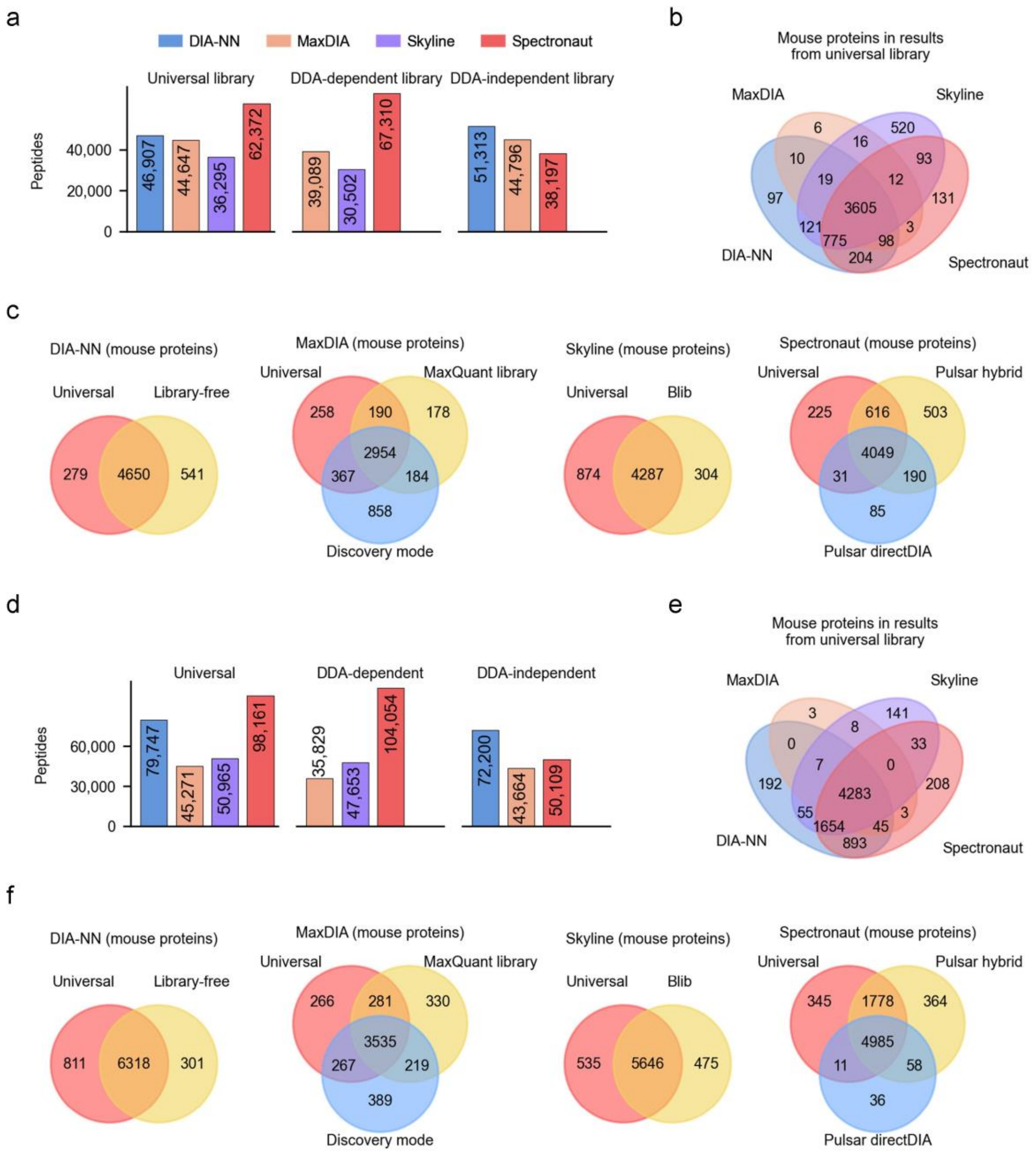

Supplementary Figure 3 Identification of mouse peptides and the overlap of mouse proteins with different analysis workflows

a, Number of mouse peptide identifications with different analysis workflows for HF data. **b,** Venn plot of proteins identified from HF data by four software tools with the universal library. **c,** Venn plots for proteins identified from HF data by four software tools with specific libraries. Re-assigned proteins based on razor protein inference are used in venn plots for cross-report comparison. **d, e, f,** Same as a, b, c but for TIMS data.

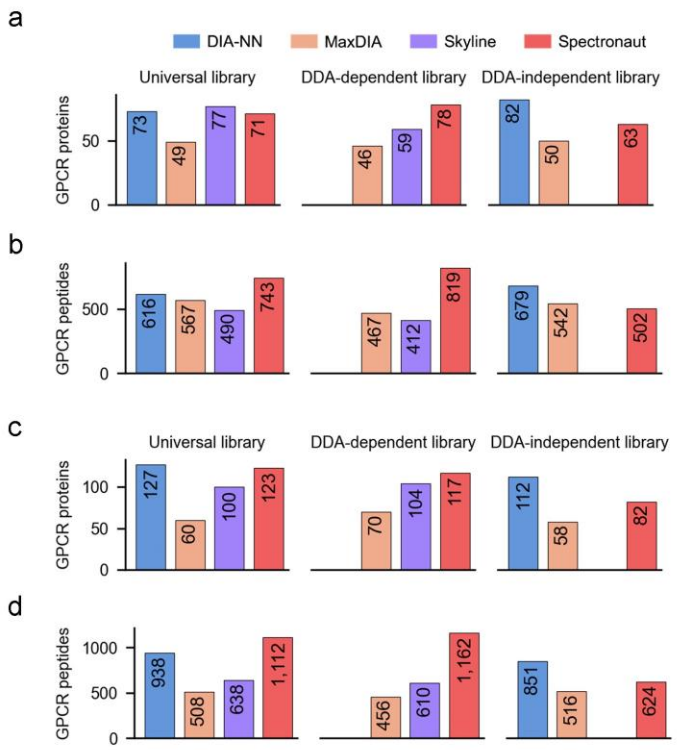

Supplementary Figure 4 Identification of GPCR proteins and peptides with different analysis workflows

a, b, Number of GPCR protein identifications **(a)** and peptide identifications **(b)** with different analysis workflows for HF data. **c, d,** Same as a and b but for TIMS data.

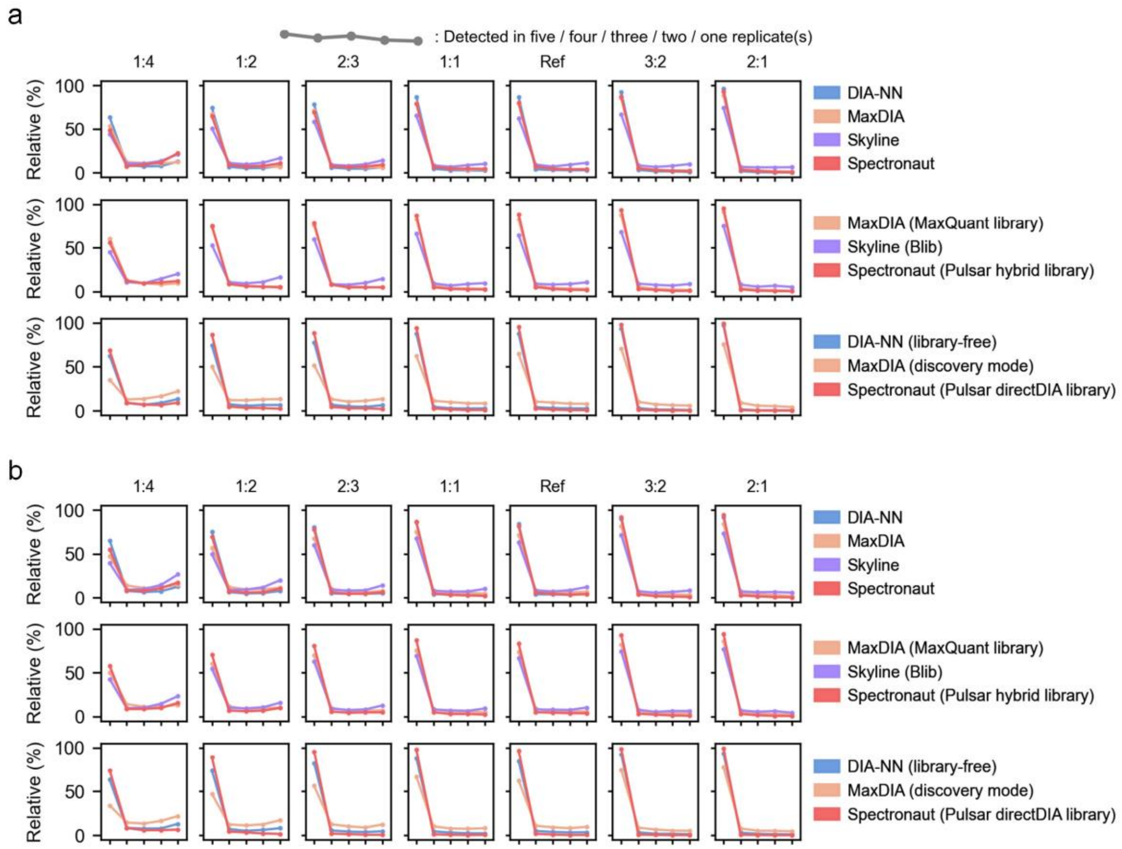

Supplementary Figure 5 Percentages of mouse proteins detected in different numbers of replicates under each dilution condition by different analysis workflows

a, Result from HF data. **b,** Result from TIMS data. Five points from left to right in each sub-figure indicate percentages of mouse proteins detected in all five, four, three, two and one replicate(s).

Source data are provided as a Source Data file at https://doi.org/10.5281/zenodo.7409391.

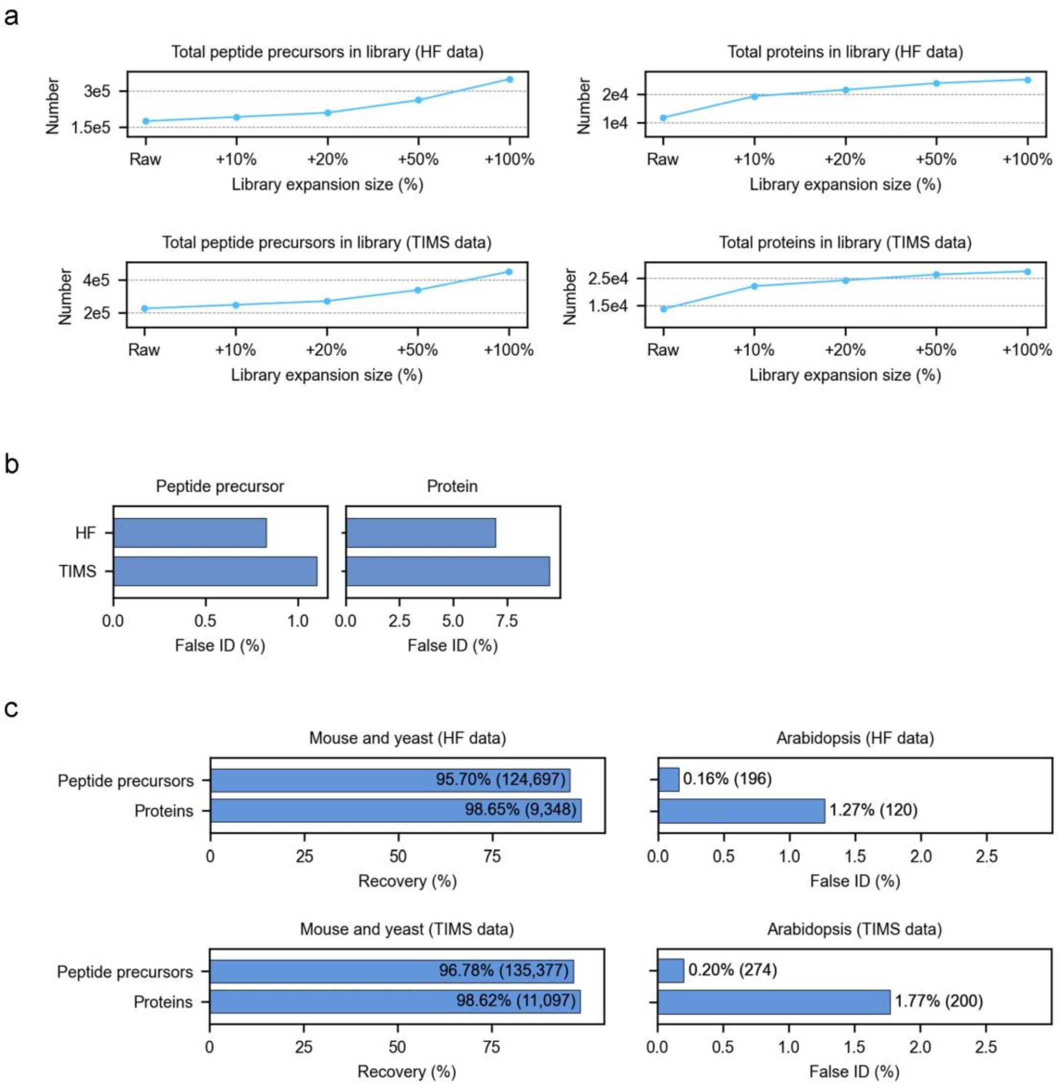

Supplementary Figure 6 Library expansion for FDR/FNR assessment

a, Number of peptide precursors (left panel) and proteins (right panel) in the target-decoy libraries incorporating increasing fractions of the Arabidopsis decoy library for the analysis of HF data (upper panel) or TIMS data (lower panel). Raw indicates the universal library serving as the target library. **b,** Percentage of false Arabidopsis peptide precursor identifications (left panel) and protein identifications (right panel) from the analysis of HF data or TIMS data by Skyline with the +10% decoy library appended. The percentage of false identifications (false ID%) was used as a proxy for FDR. **c,** Recovery% of mouse and yeast proteome (left panel) and false ID% of Arabidopsis proteome (right panel) in the analysis of HF data (upper panel) or TIMS data (lower panel) by DIA-NN with an *in silico* target-decoy library. This library was generated by merging mouse, yeast, and Arabidopsis

FASTAs. Absolute numbers of identified proteins or peptide precursors are indicated in parenthesis. Source data are provided as a Source Data file.

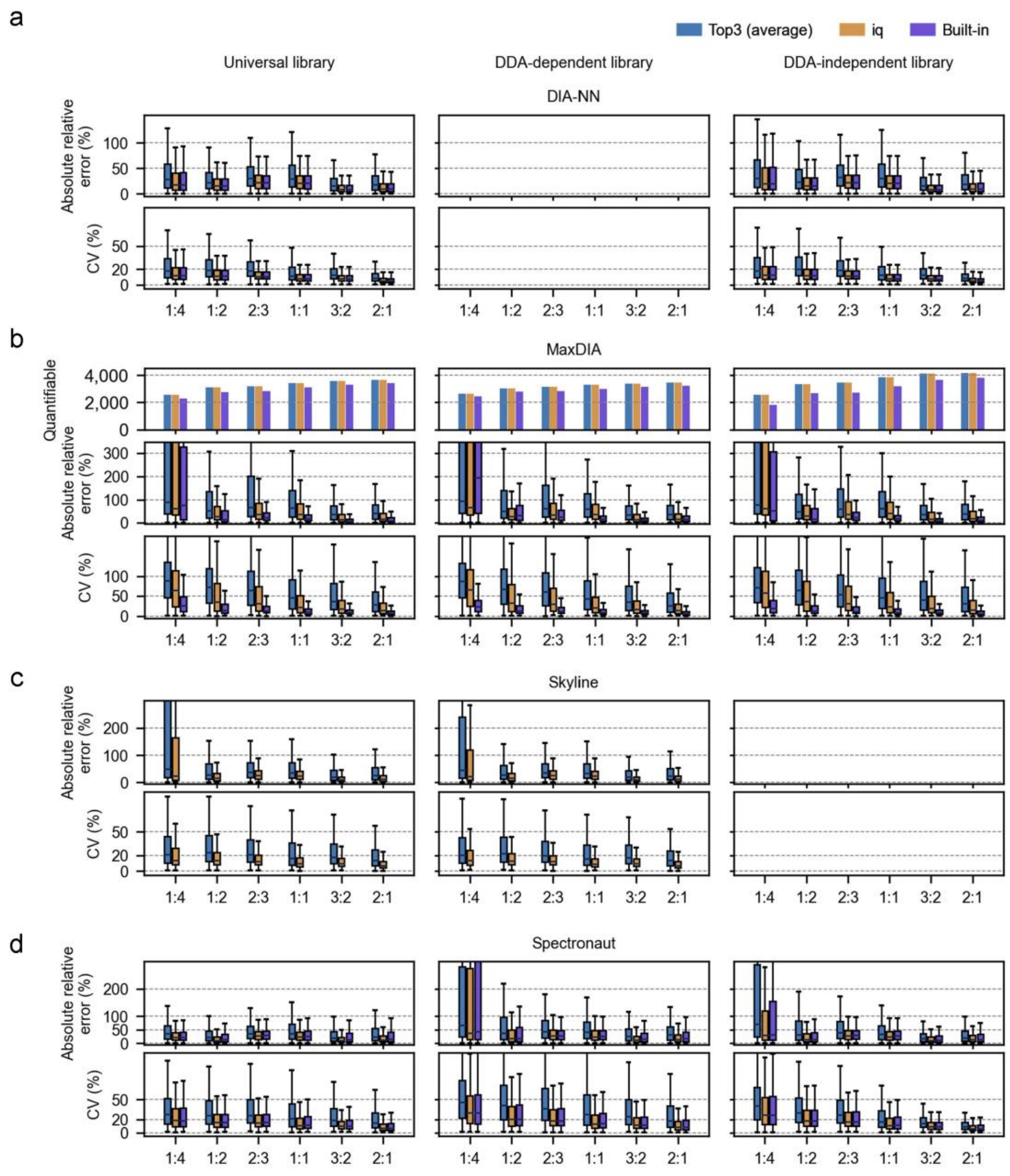

Supplementary Figure 7 Comparison of three methods for protein intensity determination by each software with specific libraries for HF data

Distribution of absolute relative errors and coefficient of variation of proteins under each condition based on quantification results by three methods (top3 average, *iq* package, and software built-in) for DIA-NN **(a)**, MaxDIA **(b)**, Skyline **(c)**, and Spectronaut **(d)**. All measurements are based on quantifiable mouse proteins which have quantities in at least 3 of 5 replicates in each condition. The quantifiable mouse proteins yielded by MaxDIA under each condition is shown in a bar plot. Boxplot center line, median; box limits, upper

and lower quartiles; whiskers, 1.5× interquartile range. Source data are provided as a Source Data file.

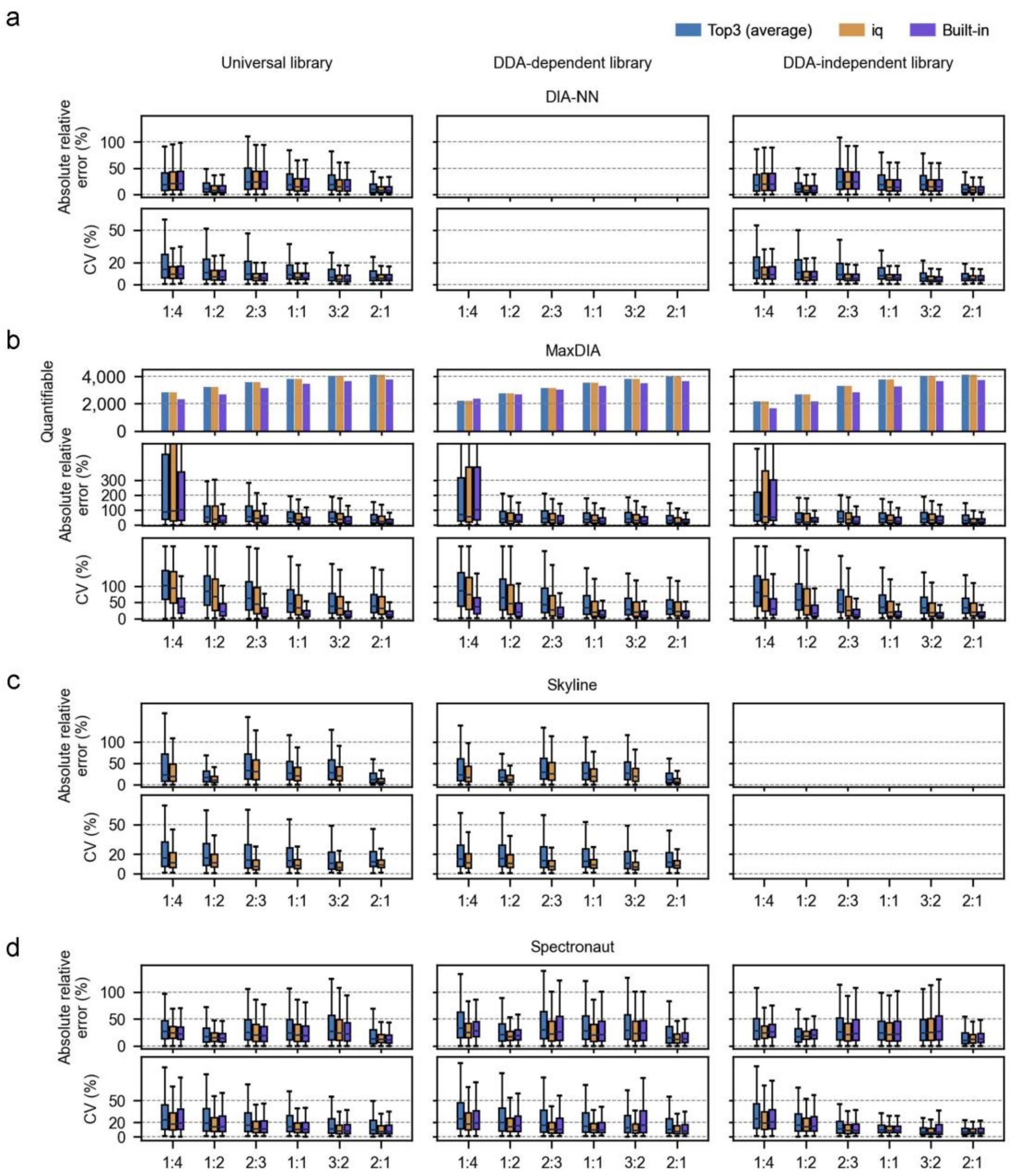

Supplementary Figure 8 Comparison of three methods for protein intensity determination by each software with specific libraries for TIMS data

Distribution of absolute relative errors and coefficient of variation of proteins under each condition (5 replicates) based on quantification results by three methods (top3 average, *iq* package, and software built-in) for DIA-NN **(a)**, MaxDIA **(b)**, Skyline **(c)**, and Spectronaut **(d)**. All measurements are based on quantifiable mouse proteins which have quantities in at least 3 of 5 replicates in each condition. The quantifiable mouse proteins yielded by MaxDIA under each condition is shown in a bar plot. Boxplot center line, median; box limits,

upper and lower quartiles; whiskers, 1.5× interquartile range. Source data are provided as a Source Data file.

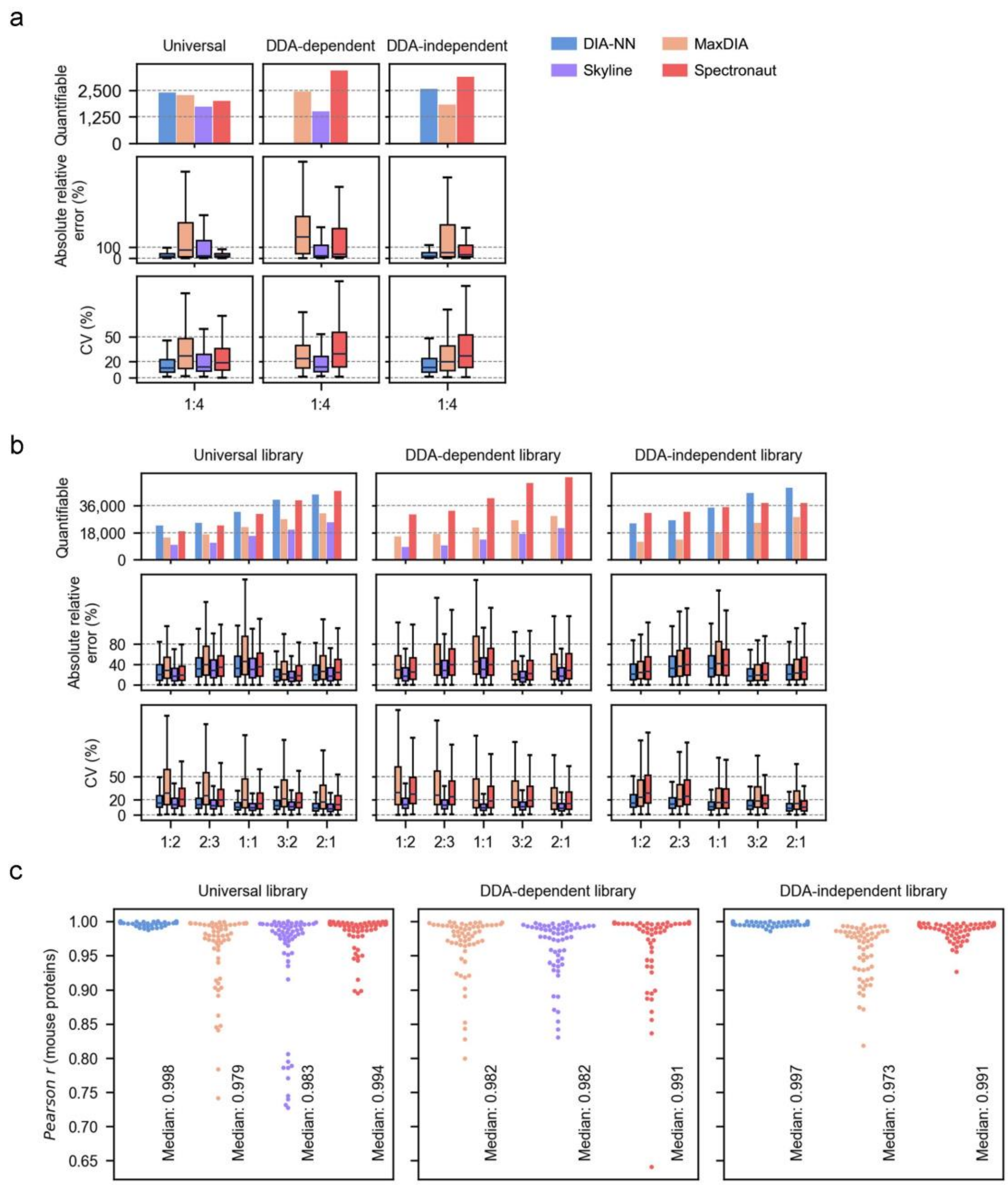

Supplementary Figure 9 Quantification performance evaluation with HF data

a, b, Protein quantification performance in condition 1:4 **(a)** and peptide quantification performance under different conditions **(b)** for HF data. Number of quantifiable mouse proteins or peptides (quantified in at least three of five replicates), distribution of absolute relative errors between expected and measured ratios, and distribution of coefficient of variation (CV) for all proteins or peptides quantified by different analysis workflows are plotted. All measurements are based on quantifiable mouse proteins or peptides which have quantities in at least 3 of 5 replicates in each condition. Boxplot center line, median;

box limits, upper and lower quartiles; whiskers, 1.5× interquartile range. **c,** Distribution of Pearson correlation coefficients (PCC) of protein intensities between replicates for different analysis workflows. Median PCC is indicated for each workflow. Source data are provided as a Source Data file.

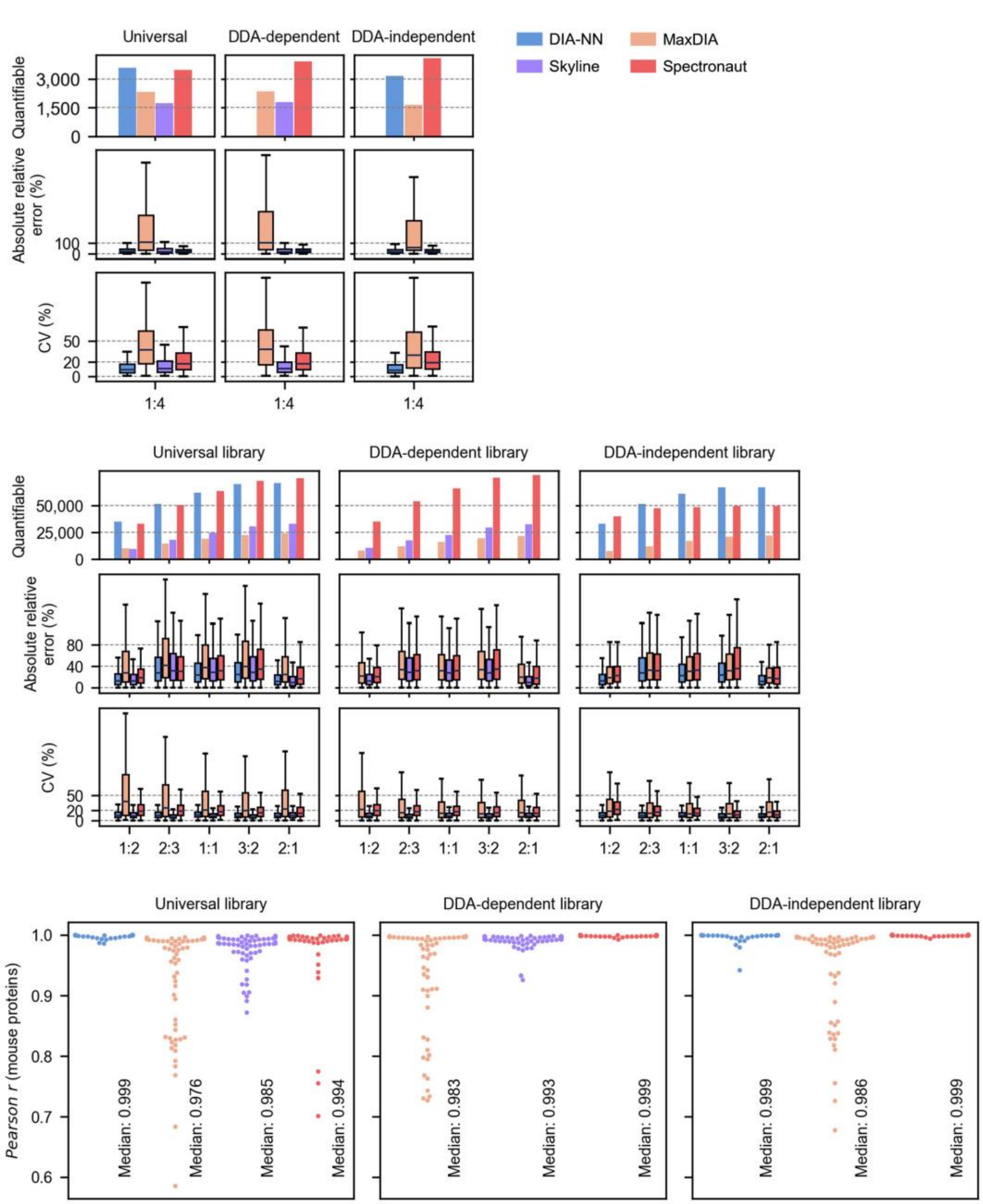

Supplementary Figure 10 Quantification performance evaluation with TIMS data

a, b, Protein quantification performance in condition 1:4 **(a)** and peptide quantification performance under different conditions **(b)** for TIMS data. Number of quantifiable mouse proteins or peptides (quantified in at least three of five replicates), distribution of absolute relative errors between expected and measured ratios, and distribution of coefficient of variation (CV) for all proteins or peptides quantified by different analysis workflows are plotted. All measurements are based on quantifiable mouse proteins or peptides which have quantities in at least 3 of 5 replicates in each condition. Boxplot center line, median;

box limits, upper and lower quartiles; whiskers, 1.5× interquartile range. **c,** Distribution of Pearson correlation coefficients (PCC) of protein intensities between replicates for different analysis workflows. Median PCC is indicated for each workflow. Source data are provided as a Source Data file.

a

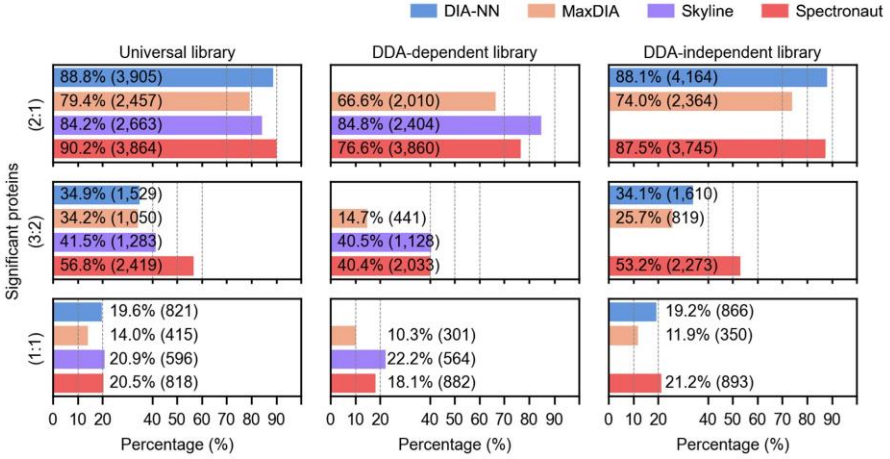

b

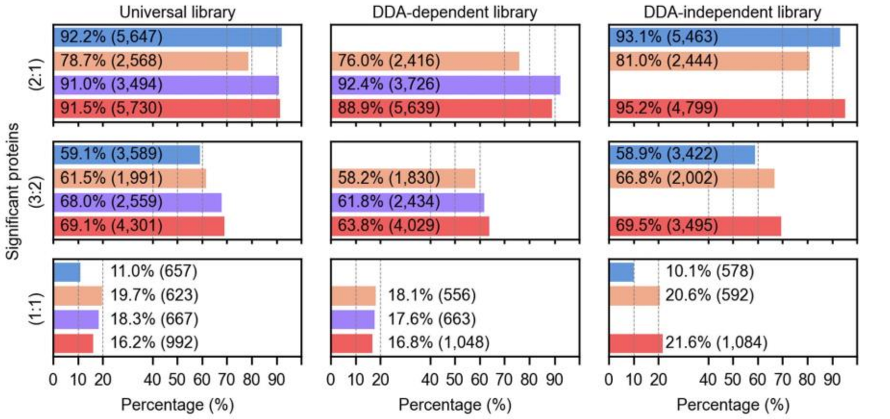

Supplementary Figure 11 Differentially expressed protein (DEP) detection

DEP detection from HF data (**a**) and TIMS data (**b**) by different analysis workflows under different conditions. Percentages of significantly changed proteins as DEPs over the total number of quantified mouse proteins in 2:1 and 3:2 conditions were used to estimate the sensitivity, while those in 1:1 condition were used to estimate the specificity. Numbers of DEPs are shown in parenthesis.

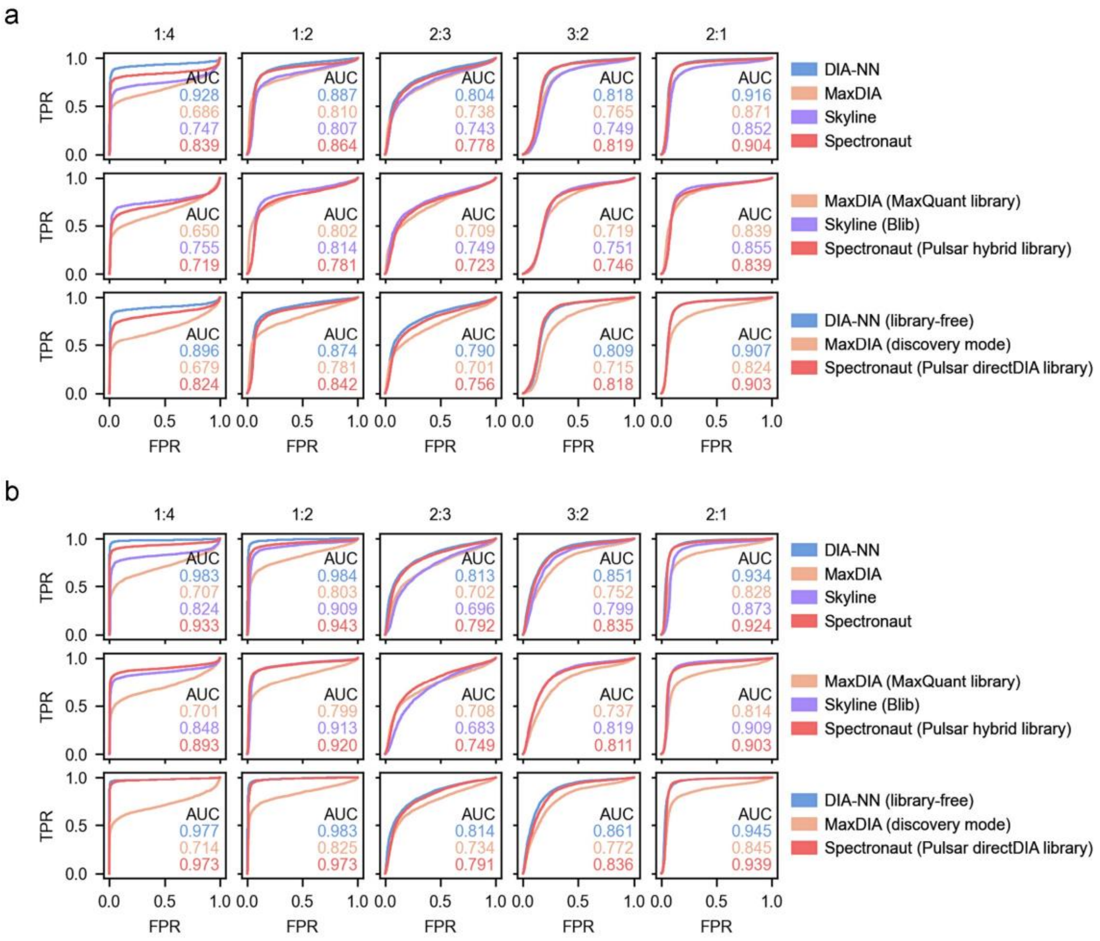

Supplementary Figure 12 Sensitivity and specificity of the DEP analysis based on receiver operating characteristic (ROC) curves

(a) HF data. **(b)** TIMS data. Area under the curve (AUC) corresponding to curves on quantification ratios is annotated for each pairwise comparison by different analysis workflows. Results are shown for specific software tools combined with the universal library (upper), software-specific DDA-dependent libraries (middle) and DDA-independent libraries (lower). Source data are provided as a Source Data file.

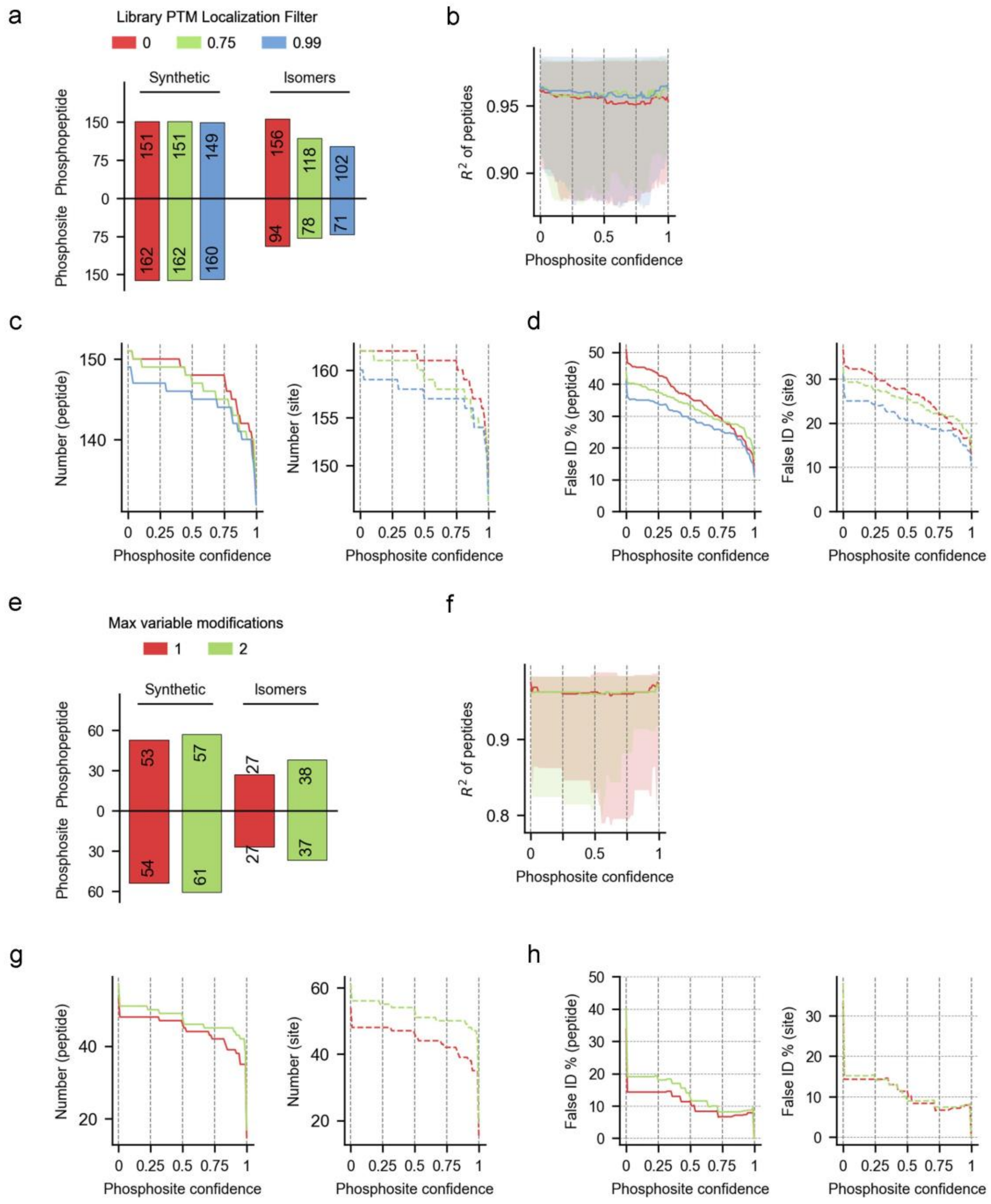

Supplementary Figure 13 Human synthetic phosphopeptide data analysis by DIA-NN and Spectronaut with an DDA-independent library

(a) Number of identified synthetic phosphopeptides and phosphosites (true hits) and isomers (false hits) in the global FDR test by Spectronaut with a directDIA library. Phosphopeptides in the library were pre-filtered using three different site localization cut-offs. **(b)** Quantification linearity of identified synthetic phosphopeptides across the dilution series by Spectronaut. The solid line indicates median values, with the interquartile range

filled in light color. **(c)** Number of phosphopeptides (left) and phosphosites (right) as a function of the phosphosite confidence score cut-off by Spectronaut. **(d)** Estimated FDR on the peptide level (left) and site level (right) as a function of the phosphosite confidence score cut-off in the global FDR test. In **(b-d)**, results are shown for the directDIA library pre-filtered using three different site localization cut-offs (red, 0; green, 0.75; blue, 0.99). **(e)** Number of identified synthetic phosphopeptides and phosphosites (true hits) and isomers (false hits) in the global FDR test by DIA-NN with the *in silico* library. Phosphopeptides in the library were pre-filtered based on its max number of phosphorylation. **(f-h)** Same as (b-d) by DIA-NN with the *in silico* library pre-filtered using the max number of phosphorylation (red, 1; green, 2). Source data are provided as a Source Data file.

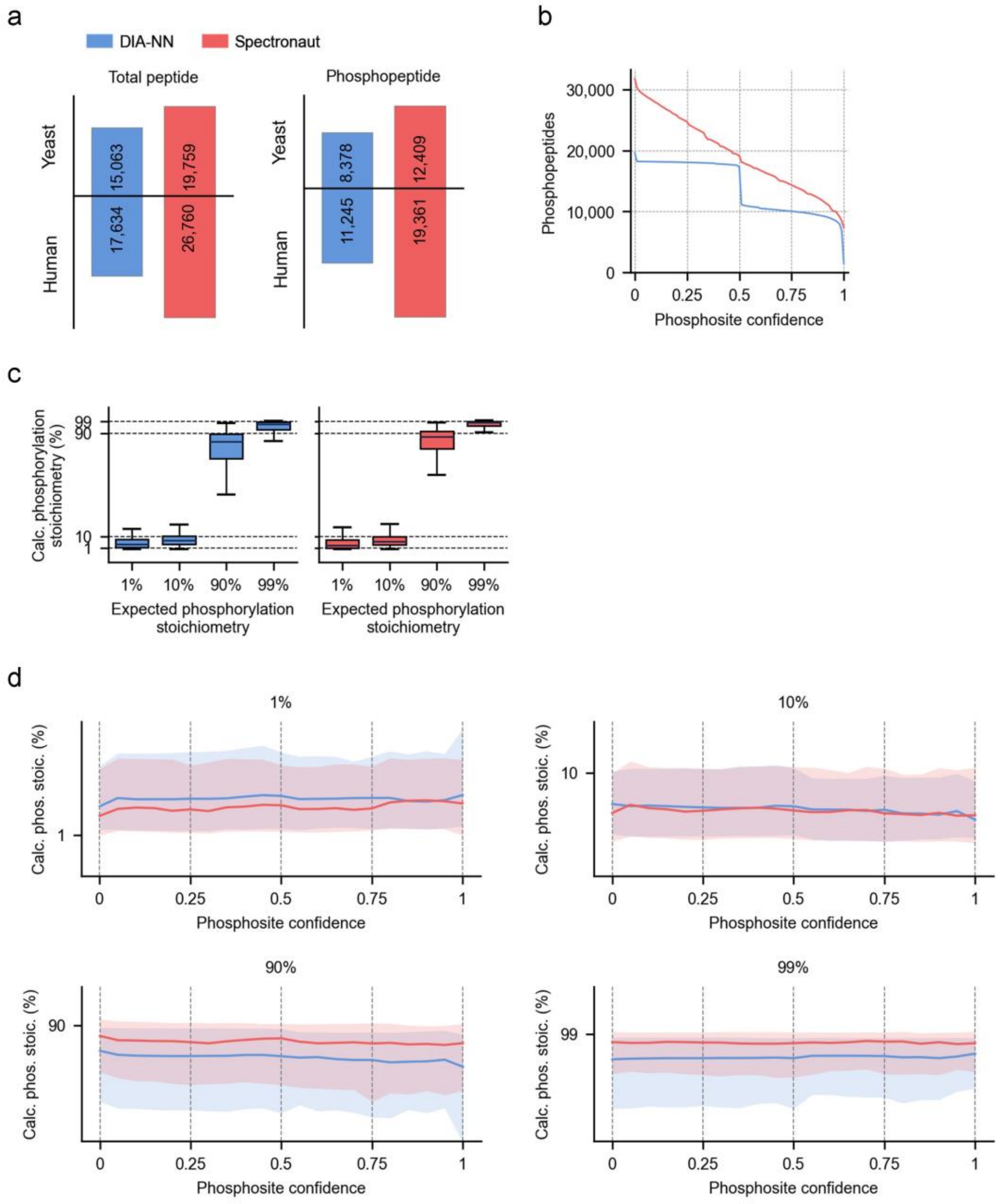

Supplementary Figure 14 Phosphorylation stoichiometry measurement using a benchmark data set.

(a) Number of identified yeast and human total peptides and phosphopeptides by DIA-NN or Spectronaut with a project-specific DDA library. **(b)** Number of identified phosphopeptides as a function of the phosphosite confidence score cut-off by DIA-NN (blue line) or Spectronaut (red line). **(c)** Boxplot of calculated phosphorylation stoichiometry based on DIA data analysis by DIA-NN (left) or Spectronaut (right). Each condition has 5 replicates, and the calculation of stoichiometry values requires the quantification matrix for a specific peptide sequence has at least 6 out of 20 runs filled.

Boxplot center line, median; box limits, upper and lower quartiles; whiskers, 1.5× interquartile range. **(d)** Distribution of calculated phosphorylation stoichiometry as a function of the phosphosite confidence score cut-off by DIA-NN (blue shade) or Spectronaut (red shade) under each condition. The solid line indicates median values, with the interquartile range filled in light color. Source data are provided as a Source Data file.

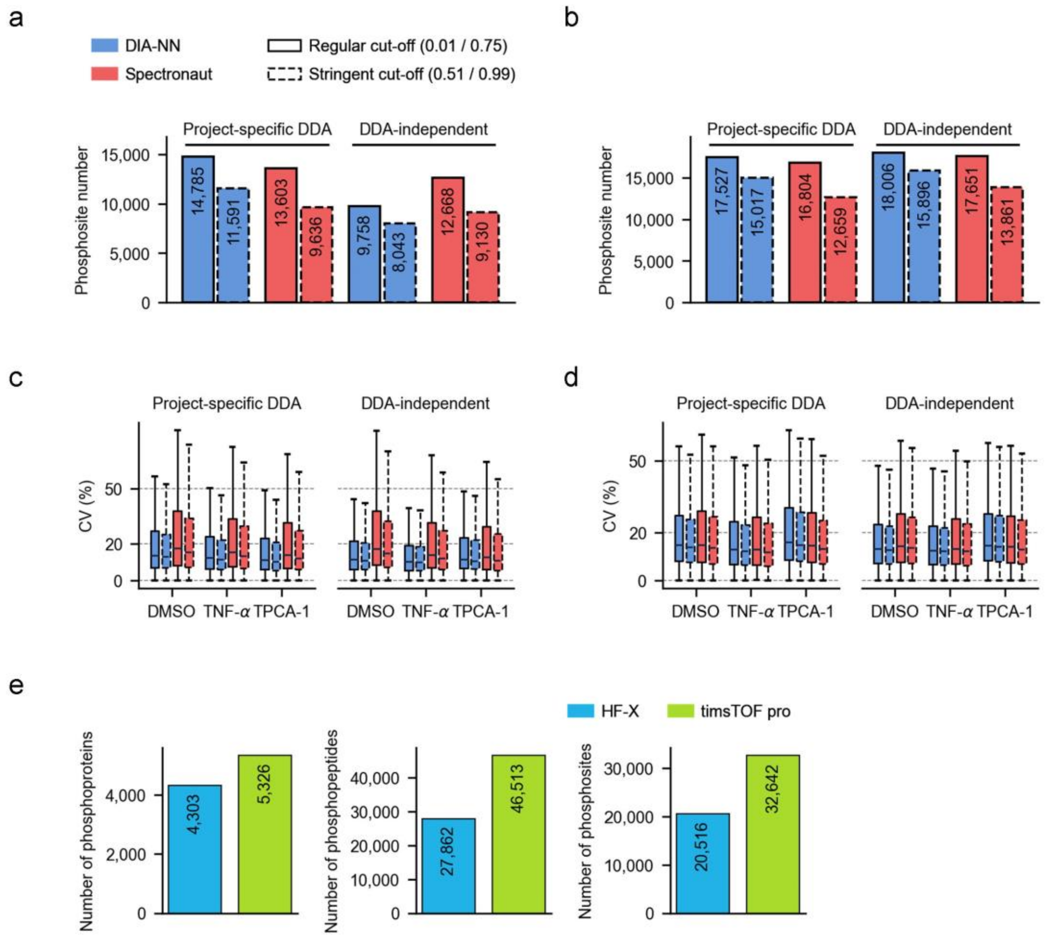

Supplementary Figure 15 Total phosphosite identification and quantification in TNF-α-induced phosphoproteomic analysis

a,b, Localized phosphosites by two software under two phosphosite confidence cutoffs from the phosphoproteome data acquired on HF-X **(a)** or timsTOF Pro **(b)**. **c,d,** Distribution of coefficient of variation (CV) for localized phosphosites under each condition for data acquired on HF-X **(c)** or timsTOF Pro **(d)**. The calculation of CV values for each phosphosite requires all 3 replicates quantified in one condition. Boxplot center line, median; box limits, upper and lower quartiles; whiskers, 1.5× interquartile range. **e,** Total phosphoproteins, phosphopeptides, and phosphosites in the FragPipe generated project-specific DDA libraries from two data sets. Source data are provided as a Source Data file.

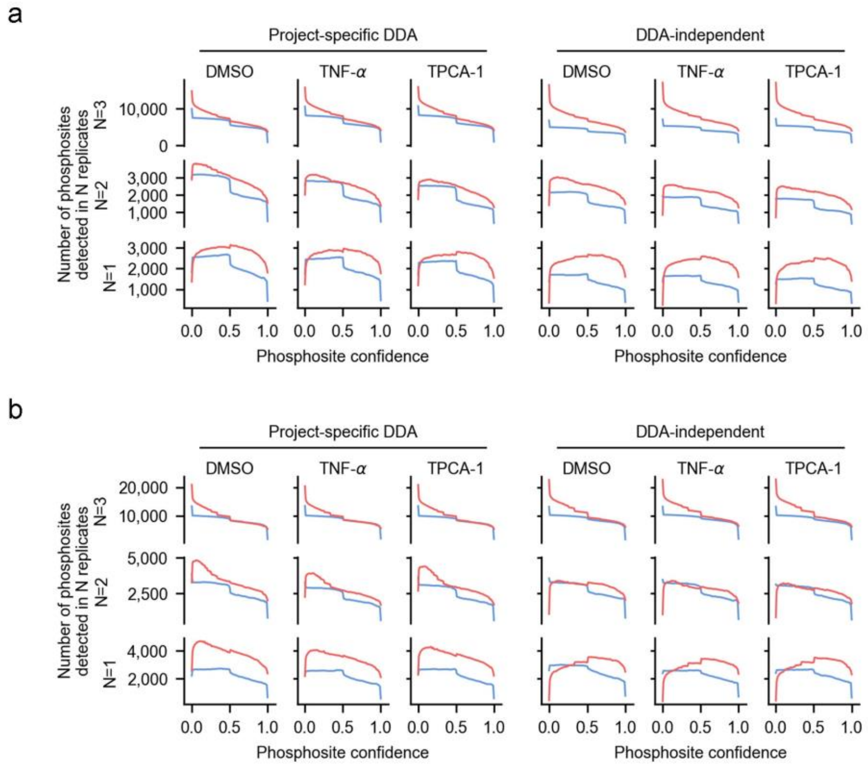

Supplementary Figure 16 Completeness of detected phosphosites in TNF-α-induced phosphoproteomic analysis without phosphosite intensity imputation. The number of phosphosites identified from N replicates at a specific condition (N=1,2,3) with DIA-NN (blue) or Spectronaut (red) is plotted as a function of the site confidence.

a, Result from HF-X data. **b,** Result from TIMS data.

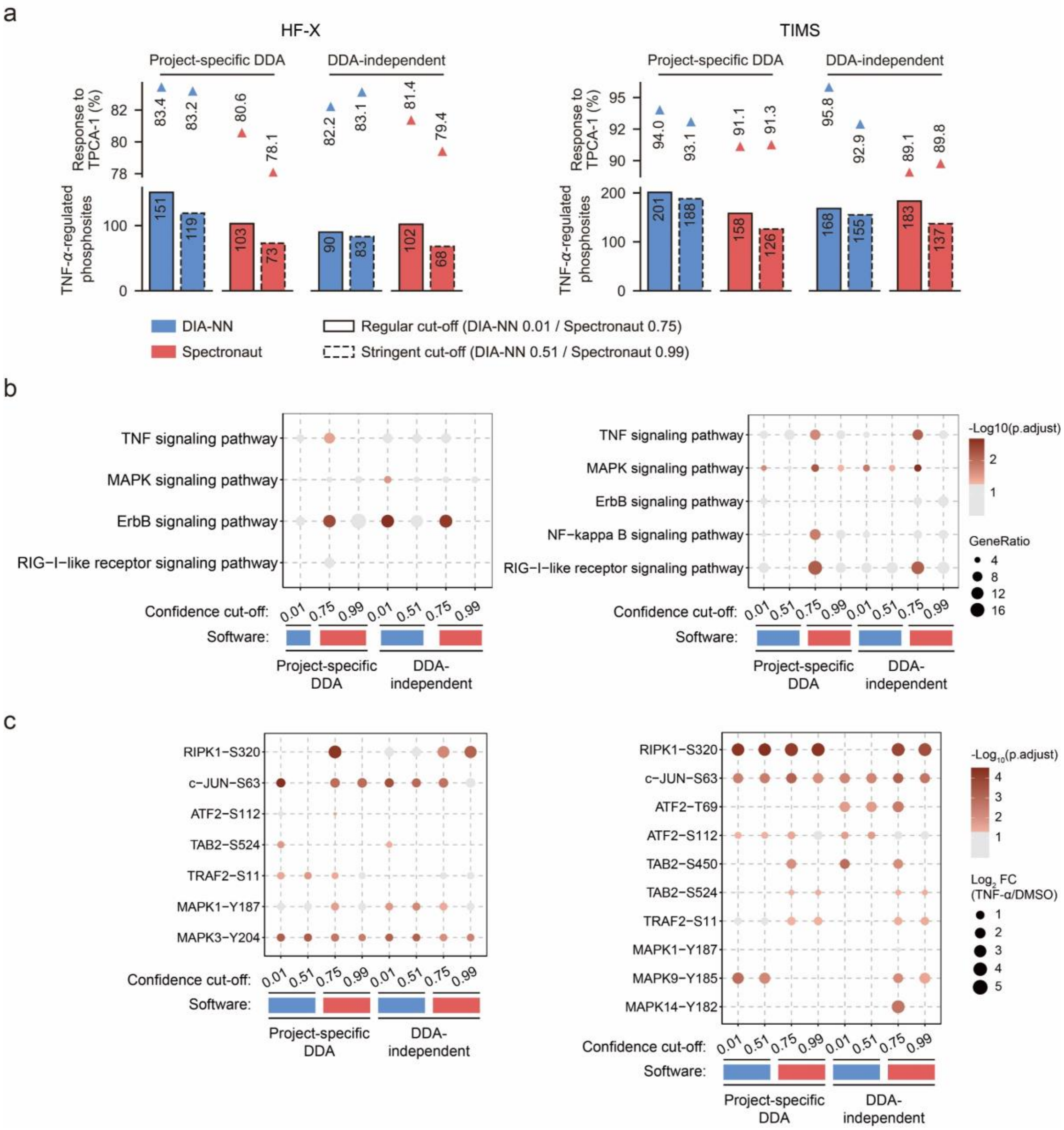

Supplementary Figure 17 Comparison of DIA-NN and Spectronaut in TNF-α-induced phosphoproteome data analysis without phosphosite intensity imputation

a, Number of TNF-α-regulated phosphosites from the analysis of HF-X data (left) and TIMS data (right) with different workflows. Phosphosites in response to TPCA-1 are those up-regulated by TNF-α and suppressed by TPCA-1 treatment, and their percentages over all up-regulated sites by TNF-α are indicated. **b,** Enriched KEGG pathways based on the analysis of HF-X data (left) and TIMS data (right) with different workflows. Significantly enriched pathways (adjusted p <0.05) are annotated in a color gradient. **c,** Phophosites up-regulated by TNF-α and included in the TNF-α pathway. They were identified in the

analysis of HF-X data (left) and TIMS data (right) with different workflows. Significantly regulated sites (FC >1.5, adjusted p <0.05) are annotated in a color gradient. Source data are provided as a Source Data file.

Supplementary Note 1 Spectronaut

In this part, we searched the two benchmark datasets using several major versions of Spectronaut (v14, v15, v16) all set with default settings (except the quantity normalization was off, and the imputation was not performed which is a default setting in v15). The exact versions we tested were 14.5, 15.4, and 16.1.

1.1 HF benchmark dataset

By analyzing the HF benchmark dataset with 3 Spectronaut versions, we see the latest version 16 generates an increased number of identified proteins, as well as the number of peptides per protein with the Pulsar generated DDA/DIA hybrid library (Supplementary Figure 18). The quantifiable identifications have a significant increase with the latest version 16, but we also observe larger absolute quantification errors and CVs in all comparisons to v14 and v15.

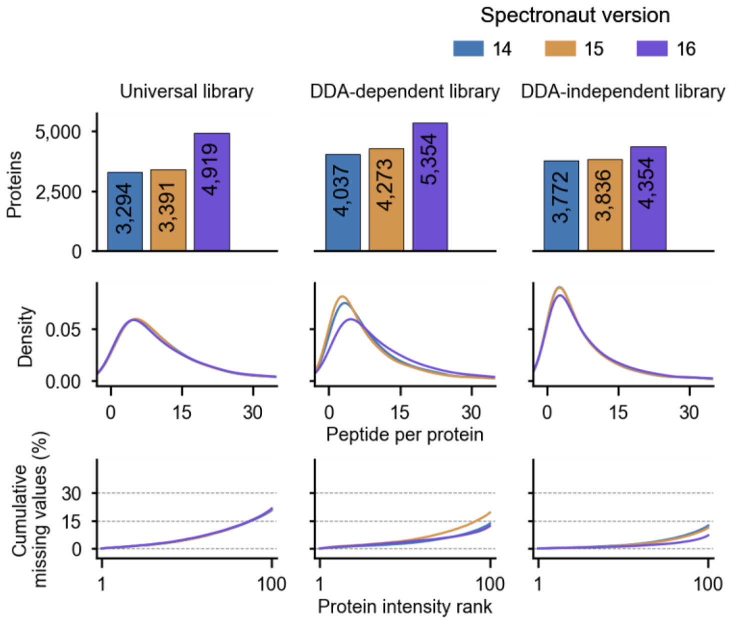

Supplementary Figure 18 Identification performance of Spectronaut in different major versions in analyzing the HF benchmark data

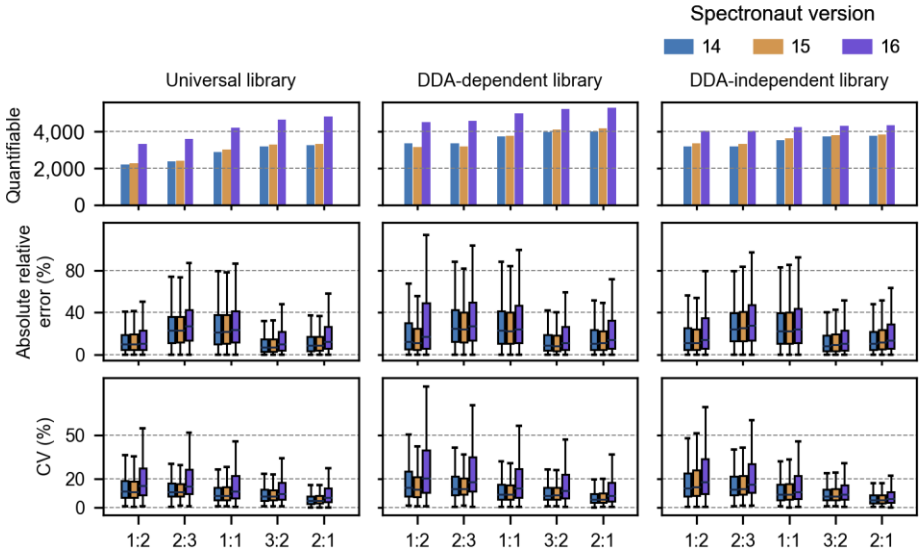

Supplementary Figure 19 Protein quantification performance of Spectronaut in different major versions in analyzing the HF benchmark data. Boxplot center line, median; box limits, upper and lower quartiles; whiskers, 1.5× interquartile range. Each condition has n=5 replicates, and quantifiable proteins are those have at least 3 quantified replicates.

1.2 TIMS benchmark dataset

Similar results can be seen from the TIMS data analysis when comparing three different versions of Spectronaut.

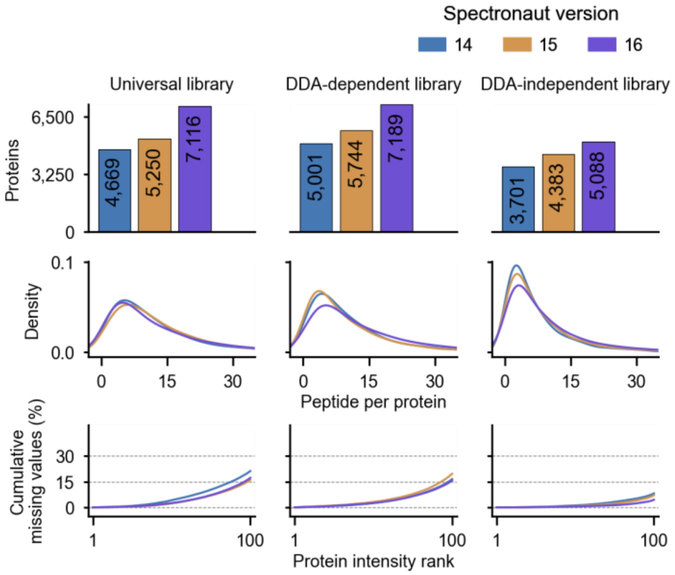

Supplementary Figure 20 Identification performance of Spectronaut in different major versions in analyzing the TIMS benchmark data

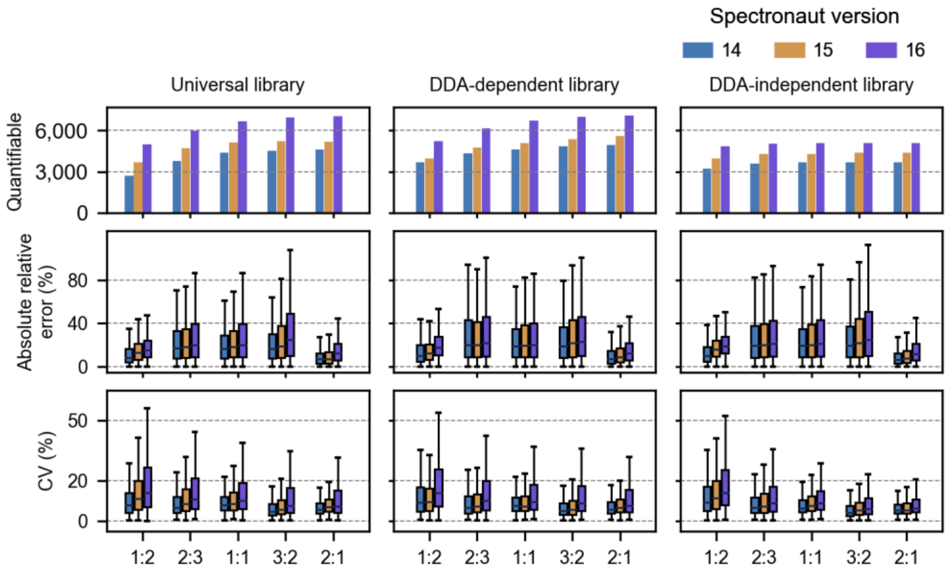

Supplementary Figure 21 Protein quantification performance of Spectronaut in different major versions in analyzing the TIMS benchmark data. Boxplot center line, median; box limits, upper and lower quartiles; whiskers, 1.5× interquartile range. Each condition has n=5 replicates, and quantifiable proteins are those have at least 3 quantified replicates.

Supplementary Note 2 DIA-NN

2.1 Empirical error rate and quantification performance with varied filtering criteria for reports from two benchmark datasets

In DIA-NN, the generally enabled match between run (MBR) option provides higher quantification completeness, with a two-step search approach by leveraging the library generated from the first pass search result and the initial library. As the developer described in the document of DIA-NN (https://github.com/vdemichev/DiaNN/blob/master/README.md), error rate control should be performed at the library level instead of the final (second pass) result when MBR is enabled. In this part, we change the filtering criteria for the reports from DIA-NN, and

estimate the empirical error rate, with four Arabidopsis peptide-appended libraries, to illustrate how much the different criteria for error rate control would affect final results.

As a pre-defined parameter, the run-level precursor q-value is set to 0.01 as default, and the further tests are based on this basic report (the long format report from DIA-NN), which is called 'Raw' in the following. While the other settings were kept as default in DIA-NN GUI (version 1.8.1, and described in methods of main text), except the classification via neural network was set to double-pass mode.

As shown in Supplementary Figure 22, we first tested 10 different filtering criteria on the report from HF benchmark data. Annotation on the top indicates q-value at what level(s) to be used, and the q-values were required to be no more than 0.01. The upper panel indicates estimated empirical false ID rate at precursor level (left) or protein group level (right). Criteria 2 - 6 are based on protein group q-value, and 7 - 10 are global-level precursor q-value or the combination of it with protein group q-values. The lower panel showed number of kept identifications under certain filtering criterion.

The criteria 1 and 7 would give higher protein group level false ID rates, as expected, since they only had precursor level FDR control. In contrast, any criterion with protein group q-value filtering (2 - 6 and 8 - 10) would give lower empirical error rates at both precursor level and protein group level. Further, any criterion with library (first pass, and with Lib annotated) protein group q-value filtering could restrict protein group error rate to lower than or approximate to 1% in the final result (second pass, and with Result annotated). And, the use of global-level protein group q-value (criterion 4 or 6) would lead to observably lower IDs, while has no observably higher power to further restrict error rate, even in the most extreme condition (+100% Arabidopsis peptides).

Since the global-level precursor q-value has no much help on further error control, we were going to find an optimal criterion among 2, 3, and 5. It’s worthy noticing that, though the criterion 3 showed about 1.6% empirical protein group error rate at the extreme condition (100% library expansion size), the other three still have good results, and, it’s very difficult to illustrate whether the additional 0.6% error rate could lead to a bad conclusion in down-stream analysis. So, it’s still worth to use the criterion 3 for real-world data. But this study focuses on the benchmarking of four tools, and the criteria 2, 3, and 5 have no such observed difference in TIMS data (Supplementary Figure 23). Here we chose criterion 2 for further analysis.

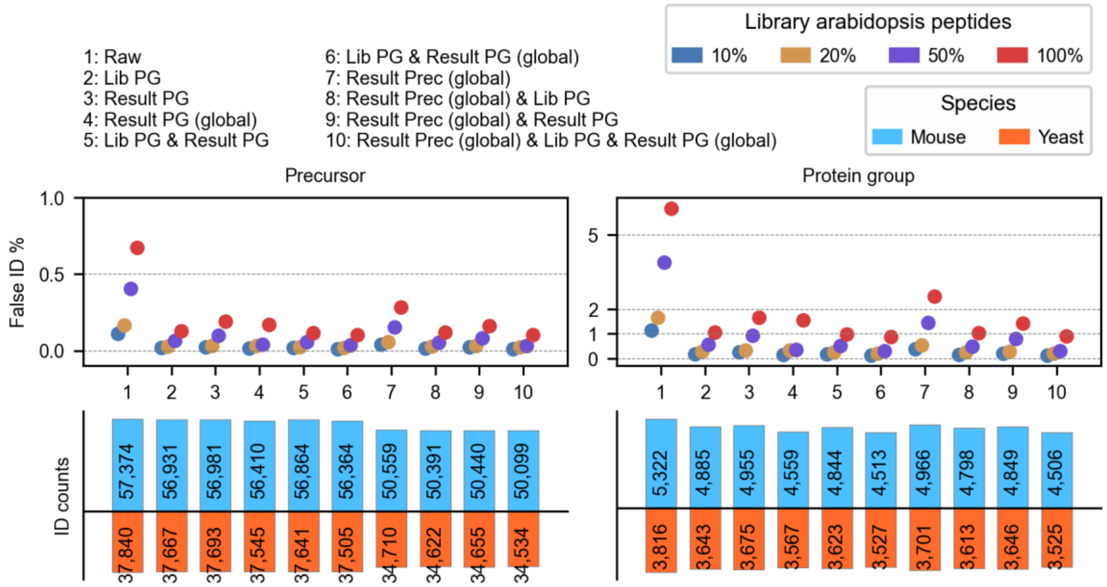

Supplementary Figure 22 Identification performance with different criteria of filtering the DIA-NN report from HF data analyzed with the universal library. Raw: exported DIA-NN long-format report with precursor level q-value filtered at 0.01. PG: protein group q-value. Lib & Result: the statistic was performed on library (the result from first pass) or final report (the result from second pass). Prec: precursor q-value. Global: the statistic was performed at global level, else at run level. All q-values were filtered at 0.01 if given.

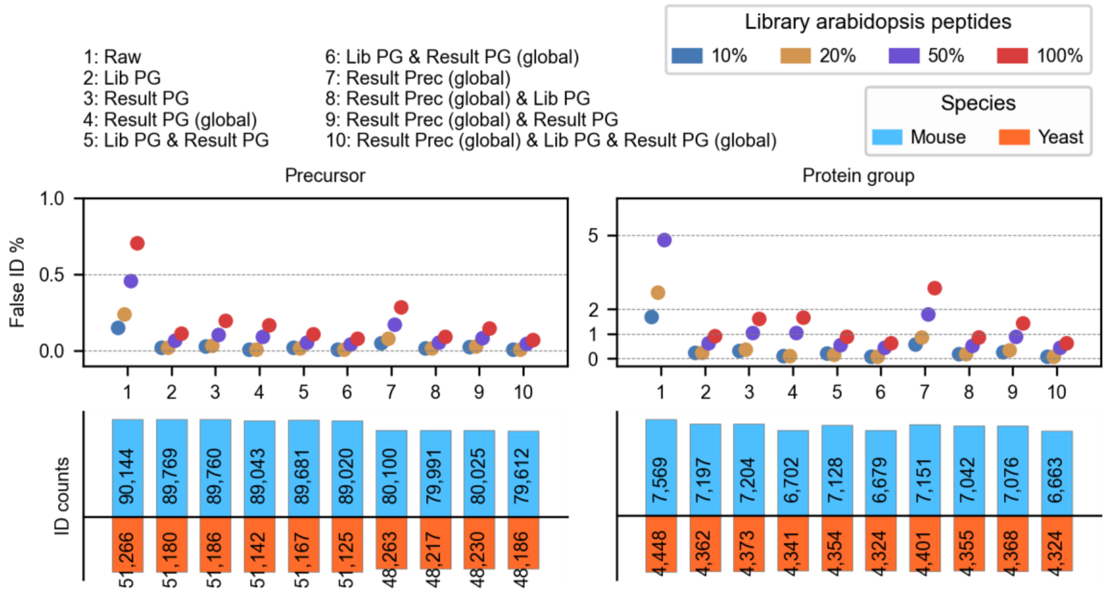

Supplementary Figure 23 Identification performance with different criteria of filtering the DIA-NN report from TIMS data analyzed with the universal library.

Besides the identification performance, we further explored the effects on quantification performance for proteins with varied filtering criteria. Supplementary Figure 24 and Supplementary Figure 25 showed the quantification results from HF data and TIMS data, respectively. These two figures have similar patterns. Here we use result from HF data (Supplementary Figure 24) as an example. Criteria 3, 5, and 9 showed better quantification accuracy and precision at the low concentration conditions (1:4 and 1:2), and have slight improvement at high concentration conditions. These indicate that, though the additional filtering for protein groups on final report (second pass) has slightly lower power than on generated library (first pass) to restrict the empirical error rate, it could improve the quantification performance, with cost of loss of some quantifiable IDs (i.e. this filtering excluded some identifications with bad quantification behavior).

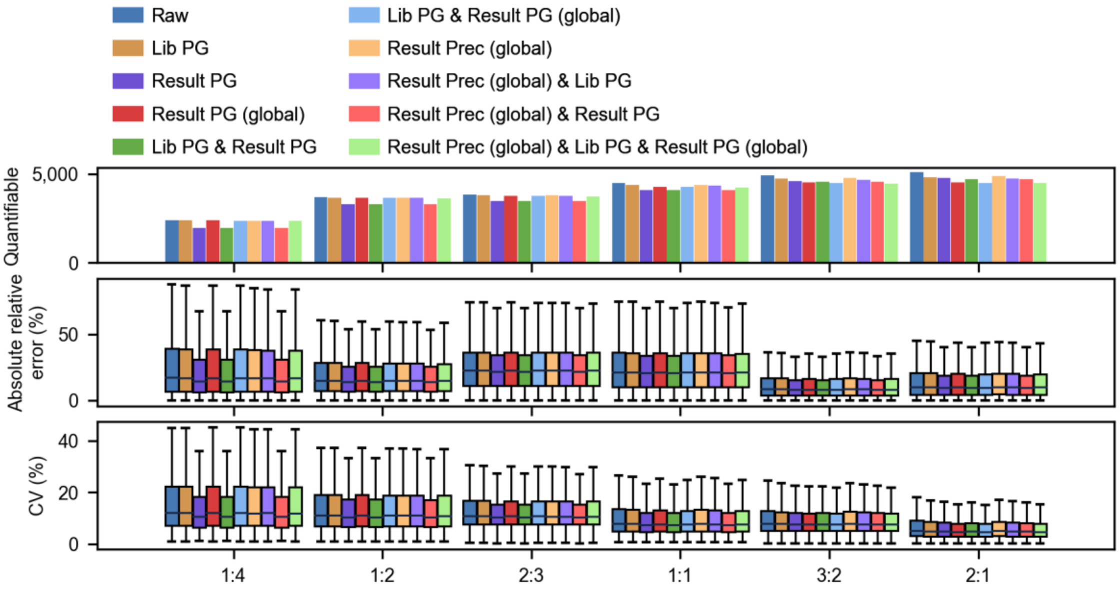

Supplementary Figure 24 Quantification performance with different criteria of filtering the DIA-NN report from HF data analyzed with the universal library. Boxplot center line, median; box limits, upper and lower quartiles; whiskers, 1.5× interquartile range. Each condition has n=5 replicates, and quantifiable proteins are those have at least 3 quantified replicates.

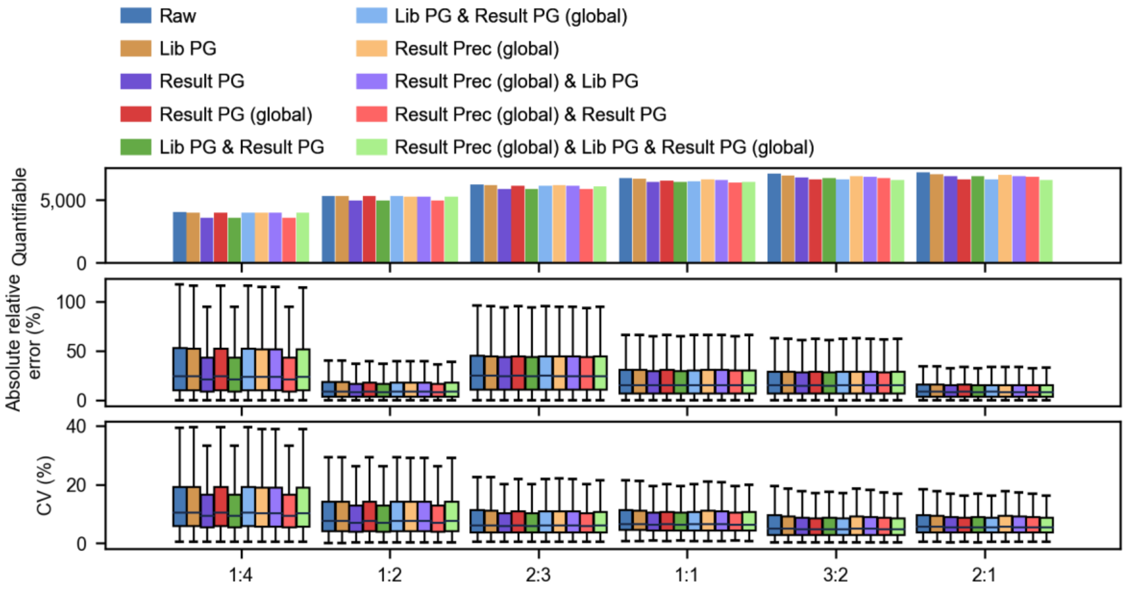

Supplementary Figure 25 Quantification performance with different criteria of filtering the DIA-NN report from TIMS data analyzed with the universal library. Boxplot center line, median; box limits, upper and lower quartiles; whiskers, 1.5× interquartile range. Each condition has n=5 replicates, and quantifiable proteins are those have at least 3 quantified replicates.

2.2 Quantification strategy

In this part, we examine to what extent the different quantification strategy would affect the quantification error and CV, with two benchmark datasets.

DIA-NN has its own protein grouping approach, and also supports an approach used in FragPipe for library generation called heuristical protein inference (enable by passing --relaxed-prot-inf to DIA-NN). Before the test of different quantification strategy settings, we first tested if the two protein grouping methods would lead to large difference on quantification performance, with the report from HF benchmark data with universal library as an example.

To avoid the potential difference at peptide level caused by different protein grouping, we didn't perform protein level error rate control in Supplementary Figure 26. And as shown in the figure, the default protein grouping approach gives more protein groups than the heuristical way in the reports, while we could get same re-assigned proteins when we do

protein grouping in the same way for these two reports, which means the reports only differ in the protein grouping methods but not in any other point. The more protein groups also lead to more quantifiable proteins, while the similar quantification performance indicates the two protein grouping approaches would not result in potential error at quantification level (Supplementary Figure 27).

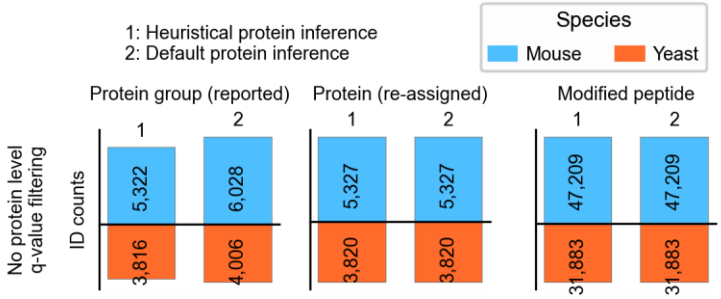

Supplementary Figure 26 Counts of identified proteins and modified peptides with two protein grouping approaches implemented in DIA-NN

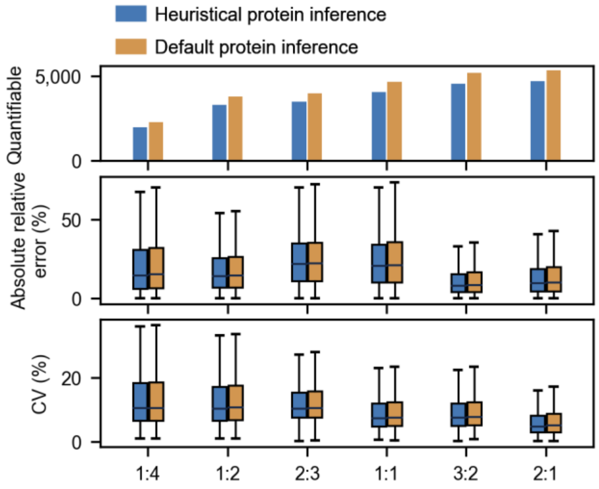

Supplementary Figure 27 Quantification performance with two protein grouping approaches implemented in DIA-NN. Boxplot center line, median; box limits, upper and lower quartiles; whiskers, 1.5× interquartile range. Each condition has n=5 replicates, and quantifiable proteins are those have at least 3 quantified replicates.

After identified the influence of identification and quantification with two protein grouping approaches, we kept protein inference with the heuristical algorithm, and tested four quantification strategies implemented in DIA-NN, for HF data (Supplementary Figure 28) and TIMS data (Supplementary Figure 29). It's not easy to determine which quantification strategy is the best with the results from two datasets, because each strategy has its preference in either accuracy or robustness of quantification. While the Robust LC with high precision would always give the best CV in all conditions and also not bad accuracy, which is chosen in this work for the data analysis in main text.

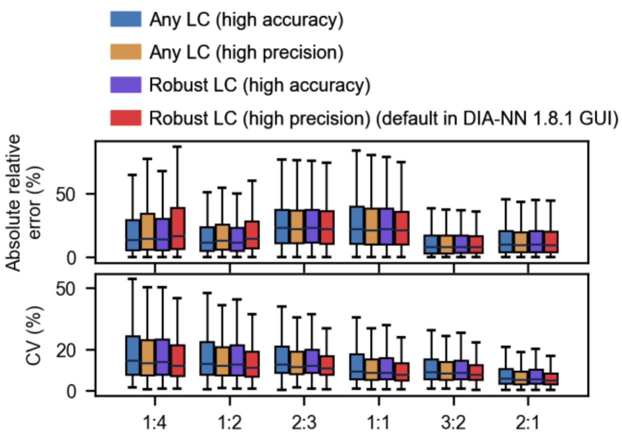

Supplementary Figure 28 Quantification performance with two protein grouping approaches implemented in DIA-NN from HF data. Boxplot center line, median; box limits, upper and lower quartiles; whiskers, 1.5× interquartile range. Each condition has n=5 replicates, and quantifiable proteins are those have at least 3 quantified replicates.

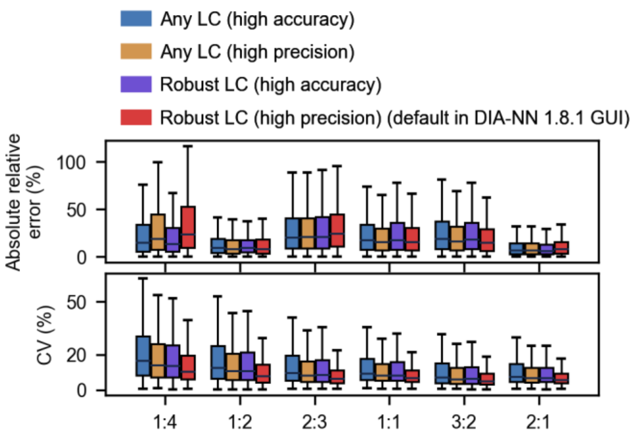

Supplementary Figure 29 Quantification performance with two protein grouping approaches implemented in DIA-NN from TIMS data. Boxplot center line, median; box limits, upper and lower quartiles; whiskers, 1.5× interquartile range. Each condition has n=5 replicates, and quantifiable proteins are those have at least 3 quantified replicates.

2.3 Filtering criteria for synthetic phosphopeptide dataset

Once DIA-NN is set to monitor a modification, it performs additional scoring for specific modified peptides. In this part, we examine what filtering criteria would be proper to keep as more as identifications and reasonable control of false error for the defined modification.

Since the monitoring of certain modification would lead to the calculation of extra sub-scores for a candidate peak group, the identification would be different with or without the requirement of monitoring a modification. As shown in Supplementary Figure 30, in all three tests, DIA-NN could report more target identifications without monitoring phosphorylation than with the monitoring. Meanwhile, the isomers would also increase without such monitoring, and the results without monitoring could be regarded as the results from other software tools such as Spectronaut with PTM cutoff of 0.

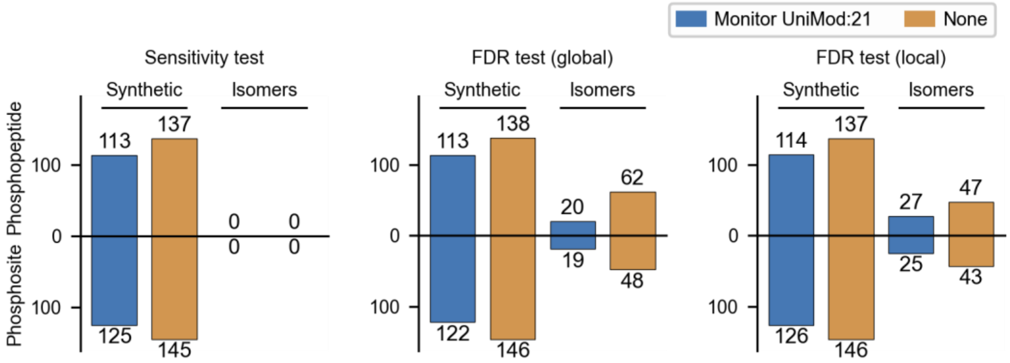

Supplementary Figure 30 Identification performance of DIA-NN by analyzing the synthetic phosphopeptide dataset with or without the requirement of monitoring phosphorylation.

By monitoring the phosphorylation, DIA-NN gives many PTM related scores in its report. In Supplementary Figure 31, we illustrated the identified targets and false ID rates along the axis of different PTM related scores. And the PTM site confidence at result level would be a good choice with good balance of number of retrieved true targets and acceptable false ID rate. The use of PTM site confidence at result level (second pass) but not the library level (first pass) as protein error control might be caused by the library was automatically filtered to have precursor q-value less than 0.01, and this potentially restricted the error of phosphopeptide at the first.

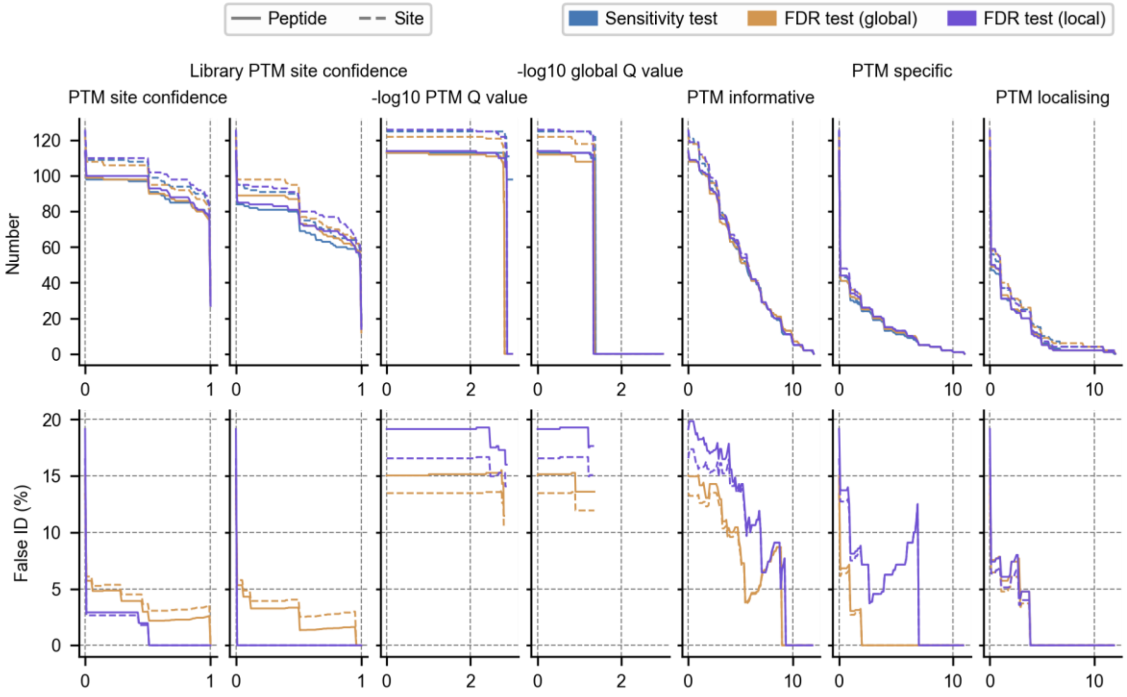

Supplementary Figure 31 Number of target identifications and estimated false ID rates along different PTM related scores reported by DIA-NN.

Supplementary Note 3 Skyline

As one of the most primary settings, the tolerance of mass error for peak matching should be determined before data searches, for specific data acquisition schemes and specific

datasets. In this note, we investigate the influence of different MS2 mass tolerance settings in the identification and quantification performance of Skyline.

3.1 HF benchmark data with different MS2 mass error tolerances

We started at a high mass error tolerance of 20 ppm for HF benchmark data, and decreased it to two common values 15 ppm and 10 ppm. Then, by calculating the sum of mean and 3-fold std from search results with 15 ppm and 10 ppm, we also used the potential optimal values 11.1 ppm and 8.6 ppm (Supplementary Figure 32). At last, a 5 ppm mass tolerance is also used to see what performance it will be.

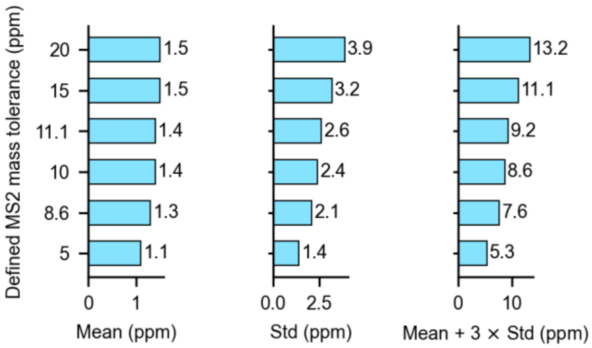

Supplementary Figure 32 The mean value and STD of mass errors from search results with defined MS2 mass tolerances in the analysis of HF benchmark data by Skyline with the universal library

With varied MS2 mass tolerance, the reports from Skyline showed an increasing trend followed by decreasing trend of identified proteins and peptides when mass tolerance decreases from a large value and reaches a very small value (Supplementary Figure 33). In the results shown here, we could see the use of 8.6 ppm as MS2 mass tolerance would lead to a gain of about 800 proteins and about 10k modified peptides. As for the quantification performance, the mass tolerance of 8.6 ppm also gives the most quantifiable identifications, with only slight increase of absolute quantification error and CV (Supplementary Figure 34). Which means a suitable mass tolerance is an important direction to adjust, and Skyline is sensitive to this parameter.

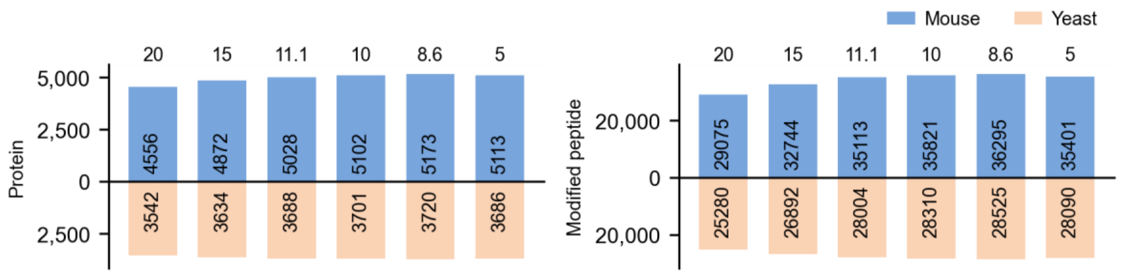

Supplementary Figure 33 Identified proteins and peptides with defined MS2 mass tolerances in the analysis of HF benchmark data by Skyline with the universal library

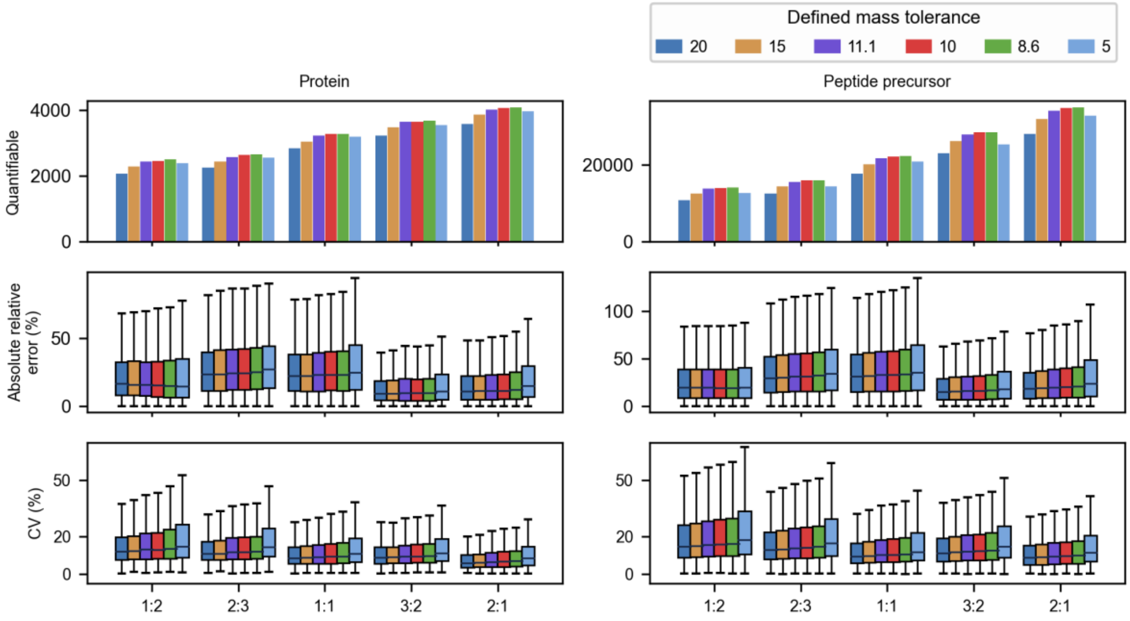

Supplementary Figure 34 Quantification performance of proteins and peptide precursors with defined MS2 mass tolerances in the analysis of HF benchmark data by Skyline with the universal library. Boxplot center line, median; box limits, upper and lower quartiles; whiskers, 1.5× interquartile range. Each condition has n=5 replicates, and quantifiable proteins are those have at least 3 quantified replicates.

3.2 TIMS benchmark data with different MS2 mass error tolerances

For TIMS data, we only viewed four values for MS2 mass error tolerance, 20 ppm, 15 ppm, 10 ppm, and 5 ppm. With same consider as HF data, 20 ppm and 5 ppm are two edges which would not be used in common cases. The 15 ppm leads to a potential optimal value 10.4 ppm, while this value is too closed to 10 ppm and we passed this value (Supplementary Figure 35). We could observe similar numbers of identifications with 4 mass tolerance, which is more stable than that from HF data (Supplementary Figure 36). While 15 ppm and 10 ppm are actually better than 20 ppm and 5 ppm. And the quantification performance shown in Supplementary Figure 37 indicates the mass tolerance of 15 ppm is the best one among these values, with the most quantifiable identifications and good quantification accuracy.

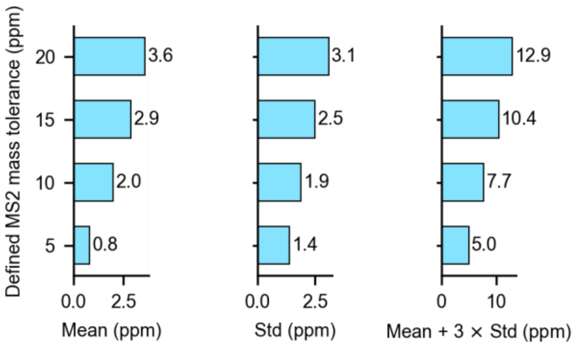

Supplementary Figure 35 The mean value and STD of mass errors from search results with defined MS2 mass tolerances in the analysis of TIMS benchmark data by Skyline with the universal library

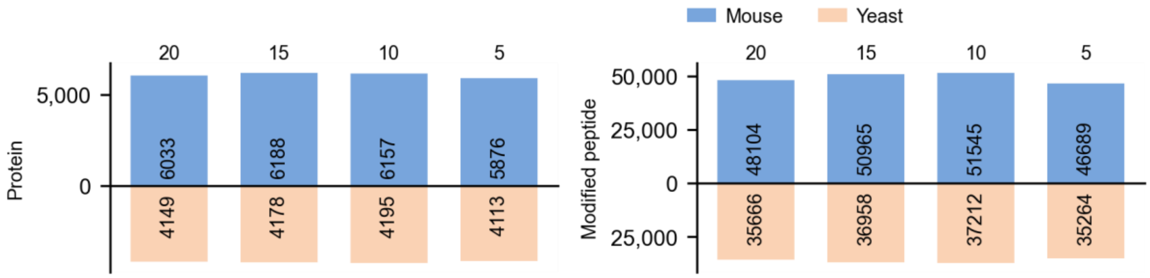

Supplementary Figure 36 Identified proteins and peptides with defined MS2 mass tolerances in the analysis of TIMS benchmark data by Skyline with the universal library

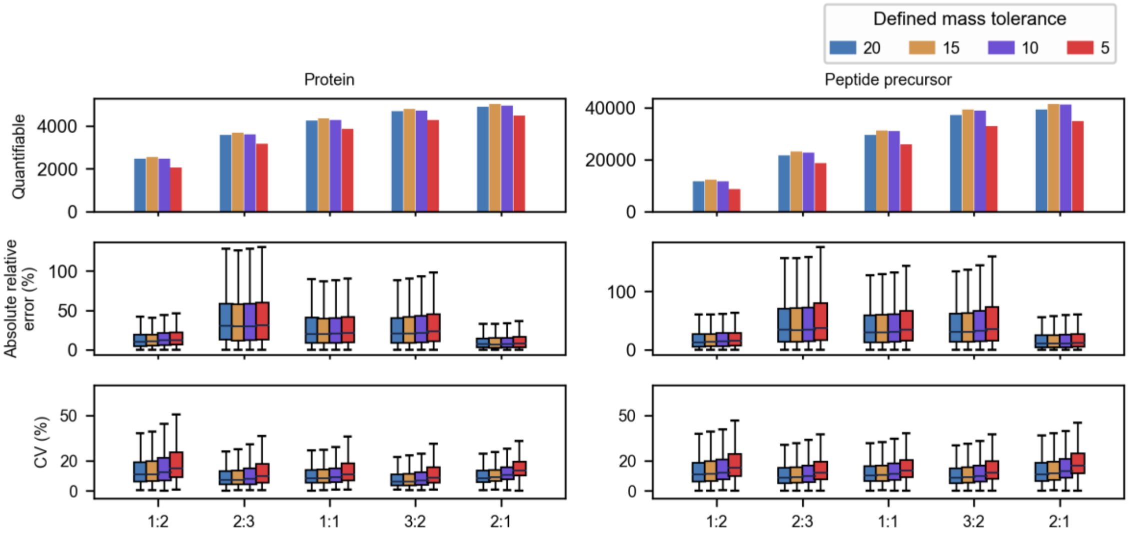

Supplementary Figure 37 Quantification performance of proteins and peptide precursors with defined MS2 mass tolerances in the analysis of TIMS benchmark data by Skyline with the universal library. Boxplot center line, median; box limits, upper and lower quartiles; whiskers, 1.5× interquartile range. Each condition has n=5 replicates, and quantifiable proteins are those have at least 3 quantified replicates.

3.3 Synthetic phosphopeptide dataset with different MS2 mass error tolerances

In this part, we further examine whether the mass tolerance could affect the identification and quantification for a relatively small dataset. We tested 4 mass tolerances in this synthetic phosphopeptide dataset (Supplementary Figure 38), and also added a combination of 5 min RT extraction window with 15 ppm tolerance, compared with 10 min used in all conditions. As shown in Supplementary Figure 39, the numbers of identified target synthetic phosphopeptides and phosphosites are the same among 4 mass tolerances, while the numbers decrease when the RT window is restricted.

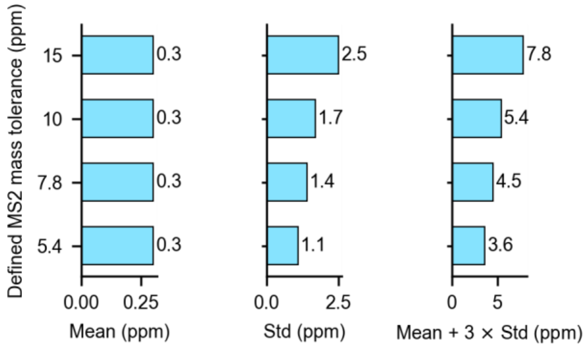

Supplementary Figure 38 The mean value and STD of mass errors from search results with defined MS2 mass tolerances in the analysis of a synthetic phosphopeptide dataset by Skyline

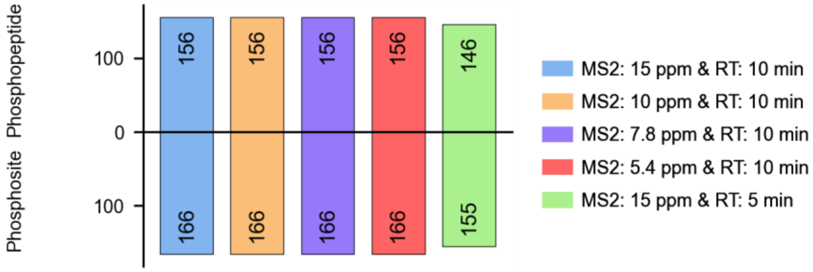

Supplementary Figure 39 Identified synthetic phosphopeptides and phosphosites with defined MS2 mass tolerances

Different quantification performance are shown in Supplementary Figure 40 among 4 mass tolerance settings. The 5.4 ppm tolerance achieved the best quantification performance. It is noteworthy that the combination of 15 ppm mass tolerance and a relatively small 5 min RT extraction window gave a quantification performance similar to the combination of 5.4 ppm and 10 min.

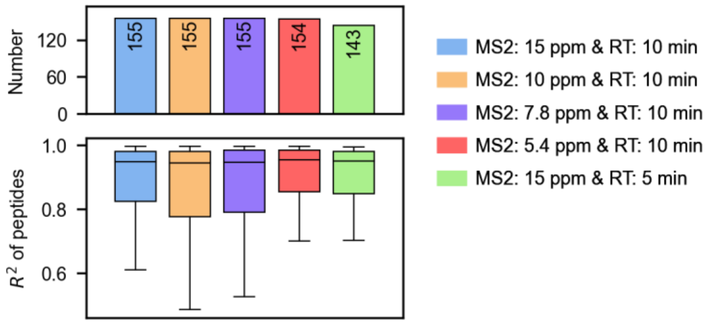

Supplementary Figure 40 Quantification linearity of identified synthetic phosphopeptides across the dilution series with defined MS2 mass tolerances and RT widths. Boxplot center line, median; box limits, upper and lower quartiles; whiskers, 1.5× interquartile range.

Supplementary Note 4 MaxDIA

4.1 Quantification performance with stepped transfer q-value

In MaxDIA, transfer q-value is an important method to increase the quantification completeness by moderating the error rate of potentially detectable precursors. Here, we scanned 5 transfer q-values to view the quantification performance under certain value. These 5 values were selected based on the scanned transfer q-values in the original paper of MaxDIA, with a narrower range. As shown in Supplementary Figure 41 and Supplementary Figure 42, we illustrated the difference of quantification performance by using quantification error (panel a) and CV (panel b). Instead of using boxplot, we show the median absolute error and median CV, and also their interquartile ranges with scatters, to directly compare these values from 5 transfer q-values. Also, we compared 3 protein quantification methods to make sure they have consistent pattern with scanned transfer q-values.

As shown in Supplementary Figure 41, we first investigated the quantification error with different transfer q-values. With a continuously decreased transfer q-value, three values, the number of quantifiable identifications, median absolute relative errors, and interquartile range of quantification error, also decreased in different degrees. In the low dilution ratio condition 1:2, the quantifiable identifications have an obvious change and quantification also become better in an obvious trend when smaller transfer q-value is used. While the

improvement is not as significant in condition 2:3 and 3:2, though there are still many losses of quantifiable identifications. And there is almost no change of quantification accuracy in condition 1:1 with lower transfer q-value and less quantifiable identifications.

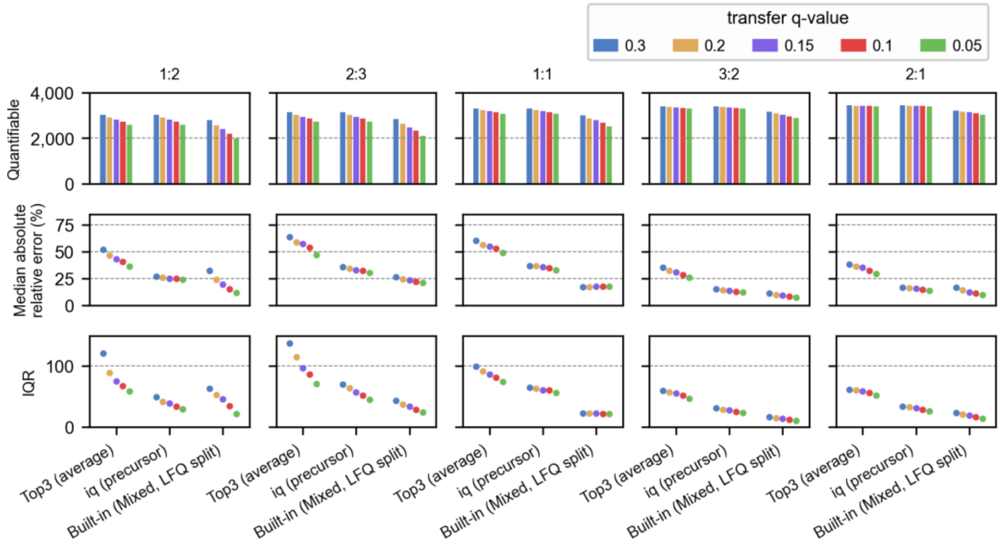

Supplementary Figure 41 Protein relative quantification error for HF data analysis by MaxDIA with stepped transfer q-values and different protein intensity determination methods

As for CV shown in Supplementary Figure 42, the built-in MaxLFQ estimated protein quantities always have stable quantification performance among replicates. Though there are certain improvements when a lower transfer q-value is used, a loss of quantifiable identifications is also observed.

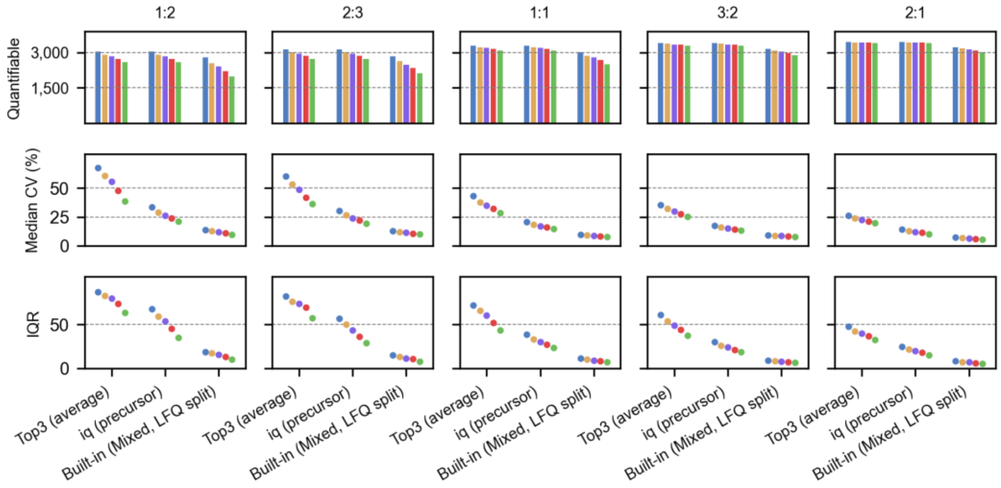

Supplementary Figure 42 Protein quantification CV for HF data analysis by MaxDIA with stepped transfer q-values and different protein intensity determination methods

The result in this part indicates one could restrict the transfer q-value to obtain more accurate quantification results, possibly at some cost of quantifiable identifications.

4.2 RT and precursor intensity correlation between MaxDIA and other tools

Here we aim to have a brief view of the correlations of peptide RT and precursor intensity among four tested software tools. As shown in Supplementary Figure 43, the bottom left part and top right part illustrate the correlations of RT and intensity, respectively. We could see the pairs of MaxDIA and other three tools have lower RT correlations, and this indicates they might have the peptides assigned to different XICs. As for the intensity correlations, we notice that MaxDIA reports relatively higher quantity values in the low intensity part, which might be related to a different method used for signal subtraction.

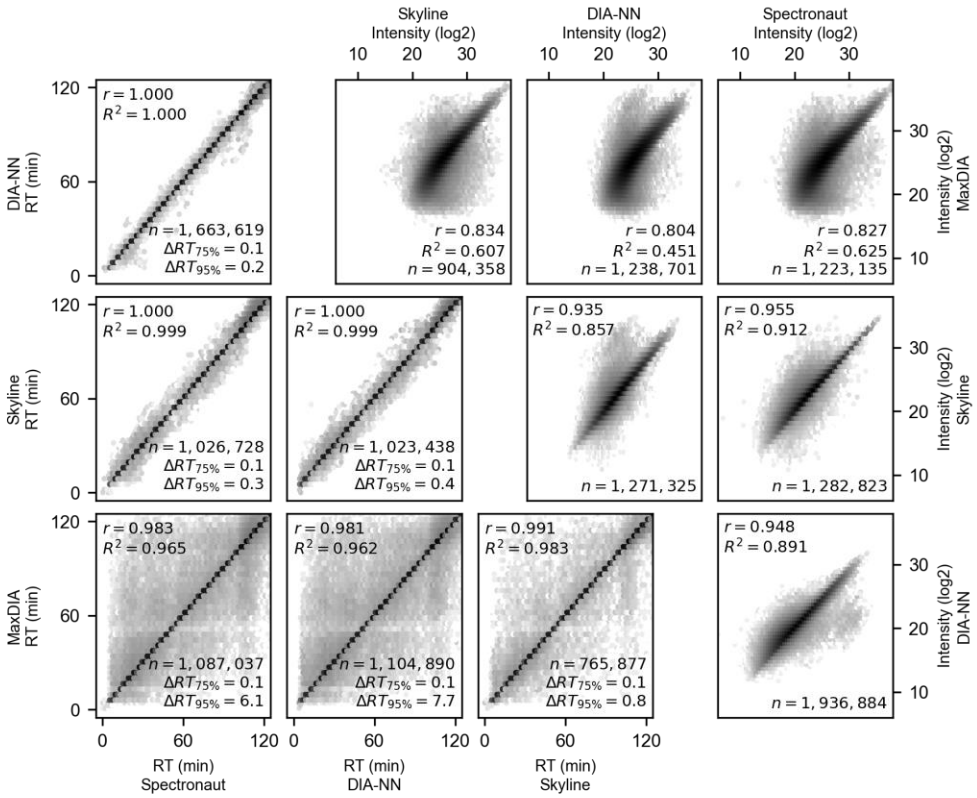

Supplementary Figure 43 Correlations of peptide RT and precursor intensity of overlapped identifications from four tested software tools in HF data analysis with the universal library

4.3 Improvement of MaxDIA in a recently released version

As a relatively new software tool for DIA data analysis, MaxDIA is continuously being developed with frequent updates. Here in Supplementary Figure 44, we show a comparison of quantification performance from MaxDIA in a previous and a latest versions in the analysis of the TIMS benchmark data with different libraries. Both quantification accuracy and precision have been improved in the latest version (2.1.3), and the total numbers of quantifiable proteins are kept at the same level.

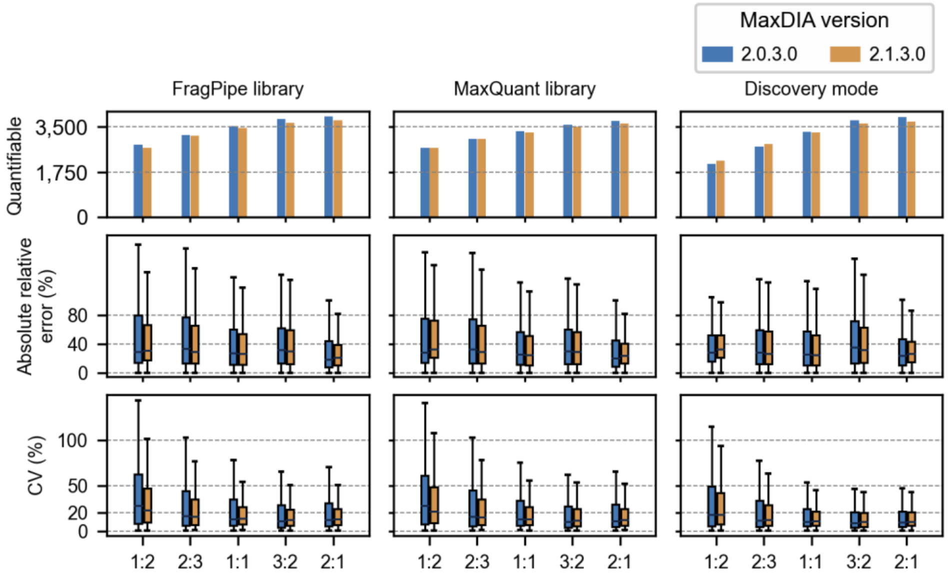

Supplementary Figure 44 Quantification performance of MaxDIA is improvement with version update. Boxplot center line, median; box limits, upper and lower quartiles; whiskers, 1.5× interquartile range. Each condition has n=5 replicates, and quantifiable proteins are those have at least 3 quantified replicates.

Supplementary Note 5 Statistics for differentially expressed protein detection

5.1 Levene's test for homoscedasticity

As a prerequisite of some statistic tests, the within-group standard deviations of the groups should be the same, that is, the homoscedasticity. We first tested if the quantified proteins among tested condition and reference condition have non-significant adjusted p-values with a Levene's test.

As shown in Supplementary Figure 45, the majority of BH-adjusted p-values from Levene's test are greater than 0.1, and other data points are also greater than 0.01.

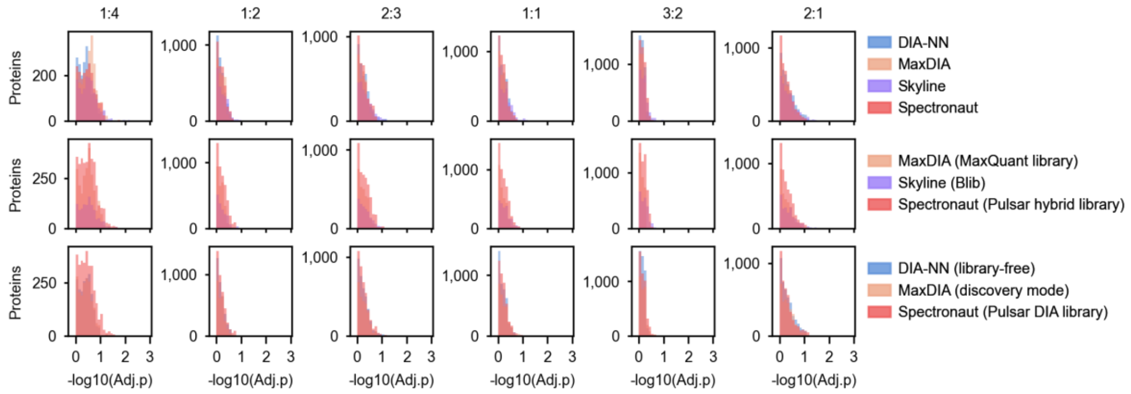

Supplementary Figure 45 Distributions of BH-adjusted p-values from Levene's test of proteins under each dilution condition by different analysis workflows for the HF benchmark data. The test is performed with mean value as center, and a proportion of 5% data points to cut from each end.

5.2 Fold-change threshold and statistical test

Generally, some modern statistic tests for differential expression detection are based on idea of the ordinary t-test (with slight modification on t-statistic value), normalization, permutation, and other strategies. For example, SAM uses a tuned s0 to ensure that the variance of statistic is independent of protein expression, and Limma moderates the standard errors across proteins with a Bayesian model. While the ordinary t-test and some t-test like methods suffer from the overestimated t-statistics caused by imprecise empirical variance on the measured abundance for protein. Here we performed the Student's t-test

on proteins among the tested conditions and reference condition in reports from different software tools, and regard a protein is differentially expressed if its BH-adjusted p-value is less than 0.05. Also, we added different FC thresholds, including 1.1, 1.2, 1.3, 1.5, and 2.0, as additional criteria for DEP detection.

As shown in Supplementary Figure 46, when no FC threshold is restricted, we retrieve the most DEPs in any dilution ratio, and this also happens in the 1:1 ratio (a negative condition, no DEPs detectable). As the FC threshold increases, the number of retrieved DEPs continuously decreases and reaches nearly 0% at a 2.0 threshold for FC. While we also see the number of DEPs correspondingly decreases in other conditions, especially when the FC threshold exceeds the theoretical dilution ratio. From this result, we choose a commonly used FC threshold of 1.5 for analysis in this study, to get a balance in sensitivity and specificity.

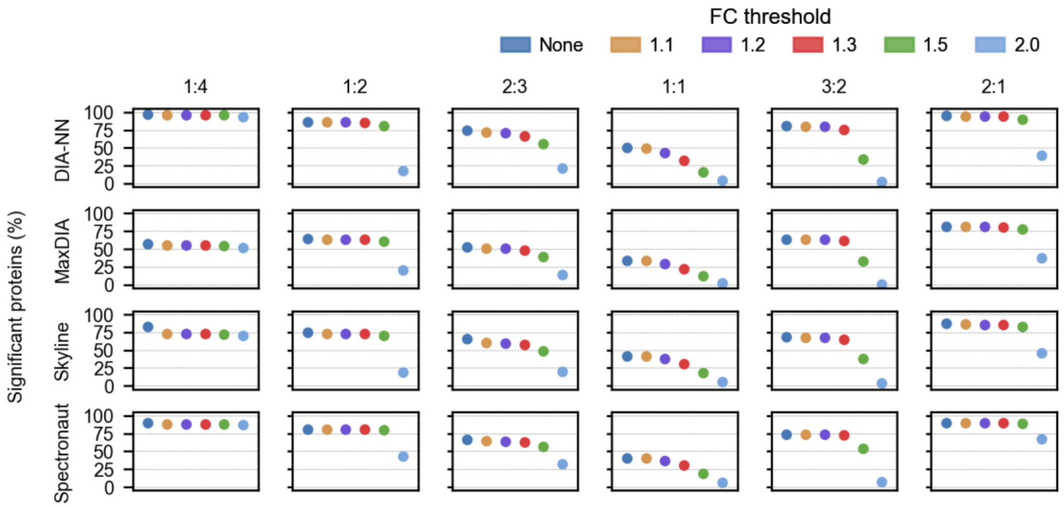

Supplementary Figure 46 Percentages of DEPs from the HF benchmark data under each dilution condition with different FC thresholds

5.3 Comparison of Student's t-test, Welch's t-test, SAM and Limma

In this part, we compare four statistical tests in the detection of DEPs in two benchmark datasets, and show their very similar performances in most conditions. We choose Limma in our data analysis.

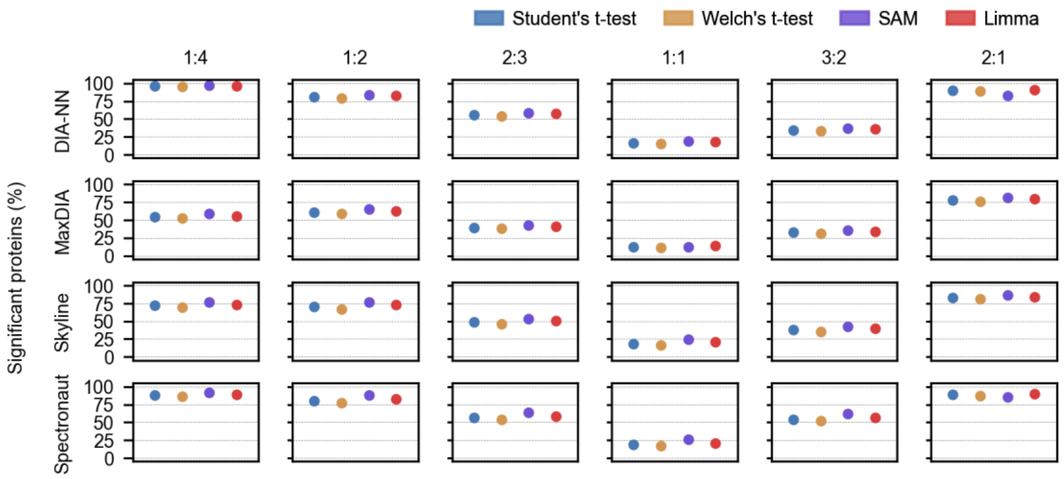

Supplementary Figure 47 Percentages of DEPs from the HF benchmark data under each dilution condition with different statistical methods
