# Supplementary material for: Benchmarking commonly used software suites and analysis workflows for DIA proteomics and phosphoproteomics
Source: Nat Commun. 2023 Jan 6;14:94. doi: 10.1038/s41467-022-35740-1 (PMC9822986; doi:10.1038/s41467-022-35740-1)
Supplement: Supplementary file 3 — Description of Additional Supplementary Files [file 41467_2022_35740_MOESM3_ESM.docx]

File Name: Supplementary Data 1

Description: Description of MS data, spectral libraries, and search results stored in iProX, and the sources of downloaded data.
