# Supplementary material for: Benchmarking commonly used software suites and analysis workflows for DIA proteomics and phosphoproteomics
Source: Nat Commun. 2023 Jan 6;14:94. doi: 10.1038/s41467-022-35740-1 (PMC9822986; doi:10.1038/s41467-022-35740-1)
Supplement: Supplementary file 5 — Reporting Summary [file 41467_2022_35740_MOESM5_ESM.pdf]

nature portfolio

Corresponding author(s): Yaoyang Zhang, Wenqing Shui

Last updated by author(s): Dec 8, 2022

Reporting Summary

Nature Portfolio wishes to improve the reproducibility of the work that we publish. This form provides structure for consistency and transparency in reporting. For further information on Nature Portfolio policies, see our Editorial Policies and the Editorial Policy Checklist.

Statistics

For all statistical analyses, confirm that the following items are present in the figure legend, table legend, main text, or Methods section.

n/a	Confirmed	
☐	☒	The exact sample size (*n*) for each experimental group/condition, given as a discrete number and unit of measurement
☐	☒	A statement on whether measurements were taken from distinct samples or whether the same sample was measured repeatedly
☐	☒	The statistical test(s) used AND whether they are one- or two-sided<br>*Only common tests should be described solely by name; describe more complex techniques in the Methods section.*
☒	☐	A description of all covariates tested
☐	☒	A description of any assumptions or corrections, such as tests of normality and adjustment for multiple comparisons
☐	☒	A full description of the statistical parameters including central tendency (e.g. means) or other basic estimates (e.g. regression coefficient) AND variation (e.g. standard deviation) or associated estimates of uncertainty (e.g. confidence intervals)
☐	☒	For null hypothesis testing, the test statistic (e.g. *F*, *t*, *r*) with confidence intervals, effect sizes, degrees of freedom and *P* value noted<br>*Give P values as exact values whenever suitable.*
☒	☐	For Bayesian analysis, information on the choice of priors and Markov chain Monte Carlo settings
☒	☐	For hierarchical and complex designs, identification of the appropriate level for tests and full reporting of outcomes
☐	☒	Estimates of effect sizes (e.g. Cohen's *d*, Pearson's *r*), indicating how they were calculated

Our web collection on statistics for biologists contains articles on many of the points above.

Software and code

Policy information about availability of computer code

Data collection	The human synthetic phosphopeptide dataset from Reta, K. et al. is downloaded from JPOST with identifier JPST000859 [https://repository.jpostdb.org/entry/JPST000859] via Mozilla Firefox.
Data analysis	FragPipe (v17.1 or v18.0) was used to process DDA data for universal library generation. Four software tools were used to process DIA-MS data or generate software-specific libraries: DIA-NN (v1.8.1), MaxDIA (v2.1.3.0), Skyline (v22.2.0.255), and Spectronaut (v16.1.220730.53000). DIATools (commit 57f3977 on 25 Mar 2021, https://github.com/cox-labs/DIAtools) was used to generate in silico library used for MaxDIA. DeepPhospho (commit a779fd9 on 5 March 2022, https://github.com/weizhenFrank/DeepPhospho) was used to generate predicted Arabidopsis library and isomeric phosphopeptide library. iq (v1.9.1) was used to estimate protein intensity. Limma (v3.50.1) was used to calculate p-values for differentially expressed protein detection. iq and Limma were used in R (v4.1.2) environment.<br>Data analysis were performed with python (3.9.7), numpy (1.12.2), scipy (1.7.1), pandas (1.3.4), dask (2021.10.0), matplotlib (3.4.3), and seaborn (0.11.2).

For manuscripts utilizing custom algorithms or software that are central to the research but not yet described in published literature, software must be made available to editors and reviewers. We strongly encourage code deposition in a community repository (e.g. GitHub). See the Nature Portfolio guidelines for submitting code & software for further information.

Data

Policy information about availability of data

All manuscripts must include a data availability statement. This statement should provide the following information, where applicable:

- Accession codes, unique identifiers, or web links for publicly available datasets
- A description of any restrictions on data availability
- For clinical datasets or third party data, please ensure that the statement adheres to our policy

Raw MS data generated in this work, spectral libraries, and MS data search reports have been deposited to the ProteomeXchange Consortium via the iProX partner repository with the dataset identifier PXD034709 [http://proteomecentral.proteomexchange.org/cgi/GetDataset?ID=PXD034709] (in ProteomeXchange) and IPX0004576000 [https://www.iprox.cn//page/project.html?id=IPX0004576000] (in iProX). The human synthetic phosphopeptide dataset from Reta, K. et al. is downloaded from JPOST with identifier JPST000859 [https://repository.jpostdb.org/entry/JPST000859] via Mozilla Firefox. All raw data, library files and search results used in this study are summarized in Supplementary Data 1.

Human research participants

Policy information about studies involving human research participants and Sex and Gender in Research.

Reporting on sex and gender	n/a
Population characteristics	n/a
Recruitment	n/a
Ethics oversight	n/a

Note that full information on the approval of the study protocol must also be provided in the manuscript.

Field-specific reporting

Please select the one below that is the best fit for your research. If you are not sure, read the appropriate sections before making your selection.

☒ Life sciences ☐ Behavioural & social sciences ☐ Ecological, evolutionary & environmental sciences

For a reference copy of the document with all sections, see nature.com/documents/nr-reporting-summary-flat.pdf

Life sciences study design

All studies must disclose on these points even when the disclosure is negative.

Sample size	Two DIA benchmark data sets generated in this work (HF data and TIMS data) have 7 conditions with 5 replicates in each. Among the 7 conditions, 1 is regarded as reference and 6 with different defined ratios of mouse and yeast peptides are used to benchmark the quantification performance of four DIA MS data analysis tools. The mouse protein ratios between samples for comparison and reference sample are defined as 1:4, 1:2, 2:3, 1:1, 3:2, and 2:1, which are sufficient to present the general situations of real-world data. The choose of 5 injection replicates also matches most of the real-cases (usually no more than 4).<br>Two datasets acquired from the TNF-α-induced phosphoproteomics experiment by QE HF-X and timsTOF Pro have 3 conditions with 3 replicates in each. The 3 replicates in these two datasets are biological replicates, and this experimental design is sufficient for downstream statistical analysis.
Data exclusions	Raw data of a public MS dataset used in this work (PXD019797, human synthetic phosphopeptide dataset) were selected from the entire dataset to ensure they are suitable for specific usages, and the finally used raw data files were listed in Supplementary Data 1. All MS data generated in this work were used without exclusion, and they were uploaded to iProX with identifier IPX0004576000 (details are listed in Supplementary Data 1).
Replication	Two DIA benchmark data sets generated in this work (HF data and TIMS data) both have 7 conditions with 5 injection replicates in each condition.<br>Two TNF-α-induced phosphoproteomics experiment data sets generated in this work (HF-X and TIMS) both have 3 conditions with 3 biological replicates in each condition.<br>The replicates from above MS data generated in this work were generated successfully.<br>The public synthetic phosphopeptide dataset has 5 conditions with 3 injection replicates in each condition, and this dataset is directly downloaded from PXD019797 with no modification.
Randomization	Not applicable. This study aims to benchmark the performance of four DIA-MS data analysis tools, and the experiment for benchmark datasets acquisition was artificially designed with no randomization, to make the benchmark datasets work as expected.
Blinding	Not applicable. There is no need to perform blinding because the results from benchmarked software are decided once the analysis is done.

Reporting for specific materials, systems and methods

We require information from authors about some types of materials, experimental systems and methods used in many studies. Here, indicate whether each material, system or method listed is relevant to your study. If you are not sure if a list item applies to your research, read the appropriate section before selecting a response.

Materials & experimental systems

n/a	Involved in the study
☒	☐ Antibodies
☐	☒ Eukaryotic cell lines
☒	☐ Palaeontology and archaeology
☐	☒ Animals and other organisms
☒	☐ Clinical data
☒	☐ Dual use research of concern

Methods

n/a	Involved in the study
☒	☐ ChIP-seq
☒	☐ Flow cytometry
☒	☐ MRI-based neuroimaging

Eukaryotic cell lines

Policy information about cell lines and Sex and Gender in Research

Cell line source(s)	Saccharomyces cerevisiae BY4742 cells were acquired from the Jilong Liu lab of School of Life Science and Technology at ShanghaiTech University. MCF-7 cells were from the Yaoyang Zhang lab of Interdisciplinary Research Center on Biology and Chemistry at Shanghai Institute of Organic. The original commercial source of MCF-7 cells is NANJING COBIOER BIOSCIENCES CO., LTD.
Authentication	Cell line was originally authenticated with PCR genotyping.
Mycoplasma contamination	Cell line was not tested for Mycoplasma.
Commonly misidentified lines (See ICLAC register)	No commonly misidentified lines were used.

Animals and other research organisms

Policy information about studies involving animals; ARRIVE guidelines recommended for reporting animal research, and Sex and Gender in Research

Laboratory animals	The C57BL/6 mice (Shanghai Jiesijie, Laboratory Animal Technology Company, China) were housed under a 12-hour light-dark cycle with ad libitum free access to water and food. The room temperature and humidity for mice were 22° to 25°C and 40 to 50%. All experimental mice were male adults (9 to 10 weeks of age) and were habituated for 1 week at least before the experiments. The mice were euthanized with 2% chloral hydrate and rapidly dissected to obtain the brain tissue.
Wild animals	The study did not involve wild animals.
Reporting on sex	Male mice were used in this study. No strict requirement for sex.
Field-collected samples	The study did not involve Field-collected samples.
Ethics oversight	The experimental procedures were approved by the Institutional Animal Care and Use Committee at ShanghaiTech University and performed in accordance with National Institutes of Health guidelines.

Note that full information on the approval of the study protocol must also be provided in the manuscript.
